# NLGP Attenuates Murine Melanoma and Carcinoma Metastasis by Modulating Cytotoxic CD8^+^ T Cells

**DOI:** 10.3389/fonc.2020.00201

**Published:** 2020-03-10

**Authors:** Avishek Bhuniya, Ipsita Guha, Nilanjan Ganguly, Akata Saha, Shayani Dasgupta, Partha Nandi, Arnab Das, Sarbari Ghosh, Tithi Ghosh, Enamul Haque, Saptak Banerjee, Anamika Bose, Rathindranath Baral

**Affiliations:** ^1^Department of Immunoregulation and Immunodiagnostics, Chittaranjan National Cancer Institute, Kolkata, India; ^2^RNA Biology and Research Laboratory, Molecular Genetics Division, CSIR-Indian Institute of Chemical Biology, Kolkata, India; ^3^Department of Zoology, Barasat Government College, Barasat, India

**Keywords:** antigen presentation, B16F10, CD8^+^ T cells, dendritic cells, LLC, metastasis, metastatic colonization, NLGP

## Abstract

Neem leaf glycoprotein (NLGP), a natural immunomodulator, attenuates murine carcinoma and melanoma metastasis, independent of primary tumor growth and alterations in basic cellular properties (cell proliferation, cytokine secretion, etc.). Colonization event of invasion–metastasis cascade was primarily inhibited by NLGP, with no effect on metastasis-related invasion, migration, and extravasation. High infiltration of interferon γ (IFN-γ)–secreting cytotoxic CD8^+^ T cells [CD44^+^, CD69^+^, GranB^+^, IFN-γ^+^, and interleukin 2^+^] was documented in the metastatic site of NLGP-treated mice. Systemic CD8^+^ T cell depletion abolished NLGP-mediated metastasis inhibition and reappeared upon adoptive transfer of NLGP-activated CD8^+^ T cells. Interferon γ-secreting from CD8^+^ T cells inhibit the expression of angiogenesis regulatory vascular endothelial growth factor and transforming growth factor β and have an impact on the prevention of colonization. Neem leaf glycoprotein modulates dendritic cells (DCs) for proper antigen presentation by its DC surface binding and upregulation of MHC-I/II, CD86, and CCR7. Neem leaf glycoprotein–treated DCs specifically imprint CXCR3 and CCR4 homing receptors on activated CD8^+^ T cells, which helps to infiltrate into metastatic sites to restrain colonization. Such NLGP's effect on DCs is translation dependent and transcription independent. Studies using ovalbumin, OVA_257−264_, and crude B16F10 antigen indicate MHC-I upregulation depends on the quantity of proteasome degradable peptide and only stimulates CD8^+^ T cells in the presence of antigen. Overall data suggest NLGP inhibits metastasis, in conjunction with tumor growth restriction, and thus might appear as a promising next-generation cancer immunotherapeutic.

## Introduction

Metastasis, an eminent hallmark of cancer (1), is an inefficacious and cumbersome expedition of tumor cells from primary to the secondary site(s) (2, 3) causing 90% cancer mortality worldwide (3). The invasion–metastasis cascade defines all the steps involved in metastasis formation; among them, successful colonization or macrometastasis formation is the last and crucial step for clinical manifestation of the disease. Disseminated tumor cells manipulate the tissue microenvironment to establish colonization successfully, including alteration in immune cell functions toward suppressive nature, as T cells and NK cells show a negative impact on colonization. Metastatic cancers illustrate low responsiveness toward classical treatments (3). Furthermore, the existence of occult systemic metastatic dissemination even before the initial detection of a primary tumor brings out extra perplexity in its treatment. Customized drugs, such as R428 (4), miR-10b antagonists (5), and Migrastatin (6), which perturb different steps of metastasis cascade (2, 7), such as invasion and migration, are incompatible for patient's treatment with already established micrometastasis (2). Hence, novel therapeutic strategies effective to act against already disseminated metastatic tumor cells (8–10) are required.

Neem leaf glycoprotein (NLGP) therapeutically restricts melanoma, carcinoma, and sarcoma solid tumor growth (11–15) by immunomodulation and increases survival of tumor host, which thus might exhibit beneficiary outcome against metastasis. To appraise this possibility, here, we had explored NLGP's antimetastatic potential against murine melanoma (B16F10) and carcinoma [Lewis lung carcinoma (LLC)]. We documented that, to combat metastasis, NLGP-mediated immunomodulation [activating CD8^+^ T cells via influencing maturation of dendritic cells (DCs)] primarily intervenes two hallmarks of cancer, that is, “avoiding immune destruction” and “angiogenesis” (1), which in turn aid to impede “colonization” (2, 7) of disseminated tumor cells at distal organs.

## Materials and Methods

### Reagents, Media, and Antibodies

RPMI-1640, Dulbecco modified Eagle medium (DMEM)-high-glucose, heat-inactivated fetal bovine serum (FBS), tri-reagent, and carboxy-fluorescein-diacetate succinimidyl ester (CFSE) were purchased from Invitrogen (Grand Island, NY, USA). CD4–fluorescein isothiocyanate (FITC)/Cy-chrome, Foxp3-phycoerythrin (PE) monoclonal antibodies (mAbs), and Cytofix/Cytoperm kit were procured from BD Pharmingen (San Diego, CA, USA). Anti-CD25 (PE), anti–MHC-I [clone 28-8-6; clone 25-D1.16; PE, FITC, and antigen-presenting cell (APC)], anti-MHC-II (PE), anti-CD80 (PE), anti-CD86 (PE), anti-CCR7 (PE), anti-CD44 (PE), and anti-CD69 (FITC) were purchased from Biolegends (San Diego, CA, USA). Purified anti–mouse transforming growth factor β (TGF-β), vascular endothelial growth factor (VEGF), and interferon γ (IFN-γ) mAbs, secondary antibodies either fluorescence or peroxidase labeled, were obtained from e-Biosciences (San Diego, CA, USA). Cytotoxicity detection kit [based on lactate dehydrogenase (LDH) release] Cyber-Green were procured from Roche Diagnostics (Mannheim, Germany). Aminoethylcarbazol (AEC) chromogen solution and aqueous mounting media were purchased from VECTOR Laboratories Inc. (Burlingame, CA, USA). Reverse transcription–polymerase chain reaction (RT-PCR) primers and cDNA synthesis kit were procured from MWG-Biotech AG (Bangalore, India) and from Fermentaus (Thermo Fisher, Waltham, MA, USA). DAPI-shield, peroxidase secondary antibodies were purchased from Sigma (St. Louis, MO, USA). Different inhibitors were procured from Calbiochem-Merck-Millipore and Sigma. CD8^+^ T cell depleting antibody (clone 2.43) was purchased from Taconic (Petersburg, NY, USA).

### Preparation of NLGP

Mature neem (*Azadirachta indica*) leaves of same size and deep green color (indicative of same age), plucked from neem trees (Geographic location of standard source: Salt Lake area of Kolkata, a City of West Bengal, India), were shed-dried and pulverized. Permission was taken to use such natural product from the National Biodiversity Authority (NBA), an autonomous and statutory body of the Ministry of Environment and Forests, Government of India (ref no. NBA/Tech Appl/9/518/12/14-15/4587). Leaf powder was soaked overnight in phosphate-buffered saline (PBS), pH 7.4; supernatant was collected by centrifugation at 1,500 revolutions/min (rpm). Crude neem leaf preparation was then extensively (four to five times) dialyzed against PBS, pH 7.4, and concentrated by Centricon membrane filter (Millipore Corporation, Bedford, MA, USA) with 10-kD molecular-weight cutoff. The purity of NLGP was checked by size exclusion–high-performance liquid chromatography (SE-HPLC) in a protein PAK 300 SW column of 7.5 mm (ID) ×30 cm. The protein peaks were determined by absorption at 280 nm in a UV recorder. Batch consistency of the NLGP is checked by HPLC and tumor growth restriction assay as per standard protocol.

### Animals

Female C57BL/6J mice (4–6 weeks; average body weight of 20–23 g) were obtained from the animal facility of CNCI, Kolkata. Autoclaved dry pellet diet (Epic Laboratory, West Bengal Government, Kalyani, India) and water were given *ad libitum*. Maintenance and treatment of animals were conducted according to the guidelines established by the Institutional Animal Care and Ethics Committee vide approval no. IAEC-1774/RB-1/2015/3.

### Cell Lines

B16F10 (melanoma) cell line was obtained from the National Center for Cell Sciences, Pune, India, and LLC (carcinoma) cell line was obtained from ATCC, Manassas VA, USA. Cells were cultured in complete DMEM high-glucose media supplemented with 10% (vol/vol) heat-inactivated FBS, 2 mM l-glutamine and penicillin (50 U/mL), and streptomycin (50 μg/mL) at 37°C with 5% CO_2_ and 95% humidity. As the cell lines were obtained from cell bank, cell authentication was not done by us.

### Spontaneous Metastasis Model and NLGP Treatment

Solid tumors were developed in C57BL/6J mice by inoculation of B16F10 or LLC (5 × 10^5^) cells subcutaneously on the right flank of mice. After palpable tumor formation, one group of mice was subcutaneously injected with NLGP (1 μg/g of body weight/injection); the other group was treated with PBS weekly for 4 weeks in total or once only as per the experimental requirement. Solid tumor growth (in mm^2^) was measured using the formula: (width × length). Tumor-bearing mice were euthanized by overdose of ketamine HCL (160 mg/kg) + xylazine (20 mg/kg) intraperitoneally (i.p.) as per Committee for the Purpose of Control and Supervision of Experiments on Animals guideline when tumor size reached 20 mm in either direction or the animal looks sick or any necrosis of tumor was occurred. The animal's health was monitored every day.

### Experimental Metastasis Model and NLGP Treatment

For the development of lung metastasis, B16F10 or LLC cells (3 × 10^5^ cells) were injected through tail-vein (t.v.). Following tumor inoculation, mice were subcutaneously injected with NLGP (1 μg/g of body weight/injection) weekly for 4 weeks in total, keeping a PBS-treated group as control.

### Histology and Immunohistochemistry

Histology and immunohistochemical analysis were performed as described (13). In brief, tissues were fixed in 10% formalin (in normal saline) for standard histological preparations and embedded in paraffin. Sections (3 μm) were prepared with microtome and stained with hematoxylin-eosin following the standard staining protocol. For immunohistochemical analysis, sections were deparaffinized with xylene and rehydrated by alcohol gradient, and then the sections were blocked with 5% bovine serum albumin (BSA) solution and stained with different primary anti–mouse antibodies overnight at 4°C. After washing with 0.01% PBS–Tween 20, sections were incubated with horseradish peroxidase (HRP)–tagged secondary antibody for 90 min. Chromogenic color was developed with AEC substrate according to manufacturer's protocol after washing with 0.01% PBS–Tween 20. Sections were counter stained with hematoxylin.

### Immunofluorescence and Confocal Microscopy

Cells were harvested by cytospin (1,500 rpm for 5 min) on poly-l-lysine–coated glass slides and fixed in methanol for 10 min (permeabilized with 0.01% Triton X after fixation). Fixed cells were stained with primary fluorescence antibody, mounted with fluoroshield-DAPI (Sigma, USA), and observed under fluorescence-microscope [LeicaDM4000B (Wetzlar, Germany) or Olympus BX53 (Shinjuku, Tokyo, Japan)] or confocal microscope (Carl Zeiss LSM800, Oberkochen, Germany).

### Western Blot

Western blot of IFN-γ, granzyme B, TGF-β, VEGF, and cleaved caspase 3 was performed as described (12). Lungs were lysed in RIPA buffer. Lung lysates (50 mg) were separated on sodium dodecyl sulfate–polyacrylamide gel and transferred onto a polyvinylidene fluoride membrane for Western blotting. Incubation was performed for different primary antibodies after blocking with 5% BSA. After washing, blots were incubated with HRP-conjugated secondary antibody for 2 h at room temperature. Western lighting chemiluminescence detection kit (Pierce, Rockford, IL, USA) was used to develop bands. Data were scanned in Quantity one (Bio-Rad, Hercules, CA, USA).

### Flow Cytometry

Flow-cytometric analysis was done as described (16). The data were generated by fluorometric analysis of 5,000, 10,000, 50,000, or 100,000 events as per experimental requirement. For cell-surface staining, cells were incubated with fluorescently labeled antibodies (specific and isotype-matched controls) for 30 min at 4°C in dark. Cells were washed twice with FACS buffer (0.1% BSA in PBS) and fixed in 1% paraformaldehyde before flow-cytometric acquisition. Intracellular molecules were stained with anti-mouse fluorescence-labeled antibodies using Cytofix/Cytoperm reagents according to the manufacturer's protocol (BD Biosciences, San Diego, CA, USA).

### Cell-Death Analysis by Annexin V–Propidium Iodide Staining

Cells were stained according to the protocol given by the manufacturer, BD Pharmingen. Cells were washed twice with cold PBS and then resuspended in 1× binding buffer. Annexin V–FITC and propidium iodide (PI) (5 μL of each) were added and mixed gently with vortexing. Cells were incubated for 15 min at room temperature (25°C) in the dark followed by resuspending the cells with 400 μL of 1× binding buffer. Cells were analyzed immediately by flow cytometry.

### Cell Cycle Analysis

Cells were harvested, fixed in chilled 70% ethanol with vortexing, followed by incubation at −20°C overnight. Cells were washed twice with PBS containing 0.1% sodium azide, followed by 50 μL (100 ng/mL) RNase treatment. Cells were stained with 200 μL (50 μg/mL) PI to analyze flow-cytometrically immediately.

### Proliferation Assay With Ki67 Staining

Ki67 staining was done as described (16). In brief, cells were harvested and fixed in chilled 75% ethanol with vortexing, followed by incubation at −20°C overnight. Cells were washed with FACS buffer twice before staining with antibody.

### Cytokine Quantification by Enzyme-Linked Immunosorbent Assay

Different protumorous cytokines from tumor conditioning media [VEGF, TGF-β, interleukin 10 (IL-10)] and cell supernatant from DC:T cell coculture (IL-2, IFN-γ) were measured (optical density at 450 nm) by enzyme-linked immunosorbent assay (ELISA) using microplate reader (BioTek Instruments Inc., Winooski, VT, USA).

### RNA Isolation, cDNA Synthesis RT-PCR, and Real-Time PCR

Cellular total RNA was isolated using Trizol (Invitrogen, Camarillo, CA, USA). Random hexamers were used to generate corresponding cDNA according to the manufacturer's protocol (First Strand cDNA Synthesis Kit; Fermentas, Hanover, MD, USA). Amplification was performed using 2× green mix (Promega, Madison, WI, USA). Amplification of the target genes was done by gene-specific primers as described (11). Primer sequence, amplification cycle, and temperature are presented in Table S1. For real-time PCR, Cyber-Green (Roche Diagnostics) was used and amplified in light cycler (Roche, Basel, Switzerland).

### *In vitro* Wound Healing Assay

A scratch was made with a scratcher on confluent B16F10 cells, followed by NLGP treatment (1.5 μg/mL). Wells were photographed at different time points to check the healing of wound (scratch).

### *In vitro* Migration and Invasion Assay

Overnight serum-starved B16F10 or LLC cells were seeded in the upper chamber of either Transwell or BD invasion chamber (4 × 10^4^ and 2 × 10^4^ cells for migration and invasion, respectively) in serum-free media in presence or absence of NLGP. Migration or invasion was measured against the 10% FBS containing media for 12 h. Following incubation, cells were fixed with 2% paraformaldehyde and stained with 0.01% crystal violet. Cells in the upper chamber were removed by wiping with cotton swabs. Serum-free gradient was used as a negative control.

### CFSE Staining, *in vivo* Migration Assay

B16F10 or LLC cells were stained with CFSE (5 mM) according to the manufacturer's protocol. Tumor (3 × 10^5^) cells were adoptively transferred through t.v. injection. Lungs were harvested at desired time points and digested with collagenase (1.5 mg/mL) and DNase I (0.1 mg/mL) for 30 min at 37°C for single-cell preparation, and CFSE^+^ cells were analyzed by flow cytometry. In a separate set, harvested lungs were prepared for cryosectioning by standard method as described (11).

### Isolation of T Lymphocytes

CD8^+^ T cells were isolated from spleen or metastatic lung (16) with the aid of positive selection using BD IMag Anti-Mouse CD8 Particles—DM (BD Biosciences). CD8^+^ T cells (>90% pure as confirmed flow-cytometrically) were either cocultured with DCs or transferred adoptively in mice.

### CD8^+^ T Cell Depletion

Tumor-bearing mice were peritoneally injected with CD8-depleting antibody (100 μg/50 μL) 24 h prior to NLGP administration on each time point. CD8^+^ T cell depletion status in peripheral blood was monitored by flow cytometry.

### Adoptive Transfer of NLGP-Activated CD8^+^ T Cells

Metastatic lungs were harvested from PBS- and NLGP-treated mice at desired time points (Figure S4DA) and digested with collagenase (1.5 mg/mL) and DNase I (0.1 mg/mL) for 30 min at 37°C for single-cell preparation. CD8^+^ T cells were isolated by magnetic bead–based positive selection (16). Isolated CD8^+^ T (2 × 10^5^) cells were adoptively transferred through t.v. injection.

### LDH Release and Antigen Restimulation Assay

CD8^+^ T cells were isolated from PBS- and NLGP-treated lungs. Cellular cytotoxicity of those CD8^+^ T cells was checked by measuring LDH release assay according to the manufacturer's protocol (Roche Diagnostics). For antigen restimulation assay, CD8^+^ T cells were restimulated, and secreted IFN-γ was measured by ELISA. Assay was performed by the method as described (15).

### Evans Blue Assay

Evans blue solution (0.1% in PBS) was injected through t.v. After 30 min of incubation, mice were sacrificed, and macroscopic observation was made.

### Generation of Bone Marrow–Derived DCs

A single-cell suspension was obtained after flushing bone marrow from tibia and femurs. Erythrocyte lysed (by ACK lysis buffer) cells (1 × 10^6^ cells/mL) were cultured with complete RPMI-1640 medium containing 10% (vol/vol) heat-inactivated FBS, 2 mM l-glutamine, and Pen-Strep (50 U/mL penicillin, 50 μg/mL streptomycin), with recombinant mouse Granulocyte-macrophage colony-stimulating factor (rmGM-CSF) (10 ng/mL) and recombinant mouse Interleukin 4 (rmIL-4) (5 ng/mL) at 37°C in 5% CO_2_ for 6 days. Media including the supplements was refreshed every third day. On day 6 of culture, non-adherent cells were harvested and considered as immature bone marrow–derived DCs (BmDCs).

### Preparation of Tumor-Conditioning Media

B16F10 or LLC cells were cultured in complete DMEM at 37°C in 5% CO_2_ up to 70% confluence. After washing, cells were cultured in fresh complete media for 48 h; exhausted media was collected and centrifuged at 2,000 rpm for 5 min, and culture supernatant was kept in aliquots at −80°C (for single use) after filtration with 0.22-μm membrane filter (Millipore, Darmstadt, Germany).

### Acid Stripping Assay for MHC-I

Acid stripping of MHC-I was done according to the method described (17). Then MHC-I surface expression status was analyzed after NLGP (1.5 μg/mL) treatment in a time-dependent manner.

### MHC-I Endocytosis Assay

Immature DCs were stained with MHC-I and then incubated at 37°C in presence or absence of NLGP, keeping controls at 4°C. After 12 h, surface MHC-I was removed by acid stripping, and internalized MHC-I was measured flow-cytometrically.

### Inhibitor Assay for Antigen Processing and Presentation Pathway

Different inhibitors MG132 (0.5 μm), chloroquine (50 μm), ammonium chloride (10 mM), brefeldin A (10 nM), cytochalasin D (10 μm), cytochalasin B (20 μm), bortezomib (1 ng/mL), leupeptine (100 μm), dimethyl amilorite (200 μm), and cycloheximide (5 μm) were used to pretreat BmDCs. B16F10, LLC lysates, ovalbumin, and SIINFEKL were also used for different assays.

### Preparation of Cell Lysates and Necrotic Cells

Viable B16F10 or LLC cells (1 × 10^7^ cells/mL) in serum-free DMEM (measured by trypan blue dye exclusion assay) were lysed by 6 or 3 freeze (in liquid nitrogen)–thaw (at room temperature) cycles for lysate or necrotic cell preparation, respectively. The lysate was centrifuged at 15,000 rpm for 30 min at 4°C, and supernatant was collected. Total protein concentration was measured using Folin's phenol reagent. Necrotic cells were centrifuged at 2,500 rpm for 5 min, and collected cells were used in different experiments.

### DC-CD8^+^ T Cell Coculture

Dendritic cells (2 × 10^5^ cells/mL), after treatment with NLGP and cell lysate (for 24 h), were fixed with 0.008% glutaraldehyde, washed with PBS, and then cocultured with autologous purified CD8^+^ T cells (1 × 10^6^ cells/mL) in a total volume of 200 μL in complete RPMI-1640 media for 7 days in a 96-well plate. Cells and supernatants were collected for further analysis.

### Software and Statistical Analysis

ImageJ (National Institutes of Health, Bethesda, MD, USA) was used for image analysis, brightness–contrast adjustment, quantification of agarose gels, manual cell counting for invasion/migration assay, and measurement of wound healing. Quantity one (Bio-Rad) was used for Western blot gel scanning and Image Studiolite (LI-COR, Lincoln, NE, USA; version 5.2.5) was used for Western blot analysis. All reported results represent the mean ± SEM of data obtained in either one (*in vivo*; *n* = 6) or three to six (for *in vitro* assays) independent experiments. Statistical significance was established by unpaired Student *t*-test (for two groups) or one-way analysis of variance followed by Tukey *post-hoc* test (for more than two groups) using GraphPad Prism 5 software (GraphPad Software, San Diego, CA, USA). For survival study, Kaplan-Meier survival analysis followed by data analysis with log-rank (Mantel-Cox) test was used. Differences between groups attaining a *p*-value of 0.05 are considered as significant.

## Results

### NLGP Attenuates Spontaneous Melanoma and Carcinoma Metastasis

**Figure 1:**
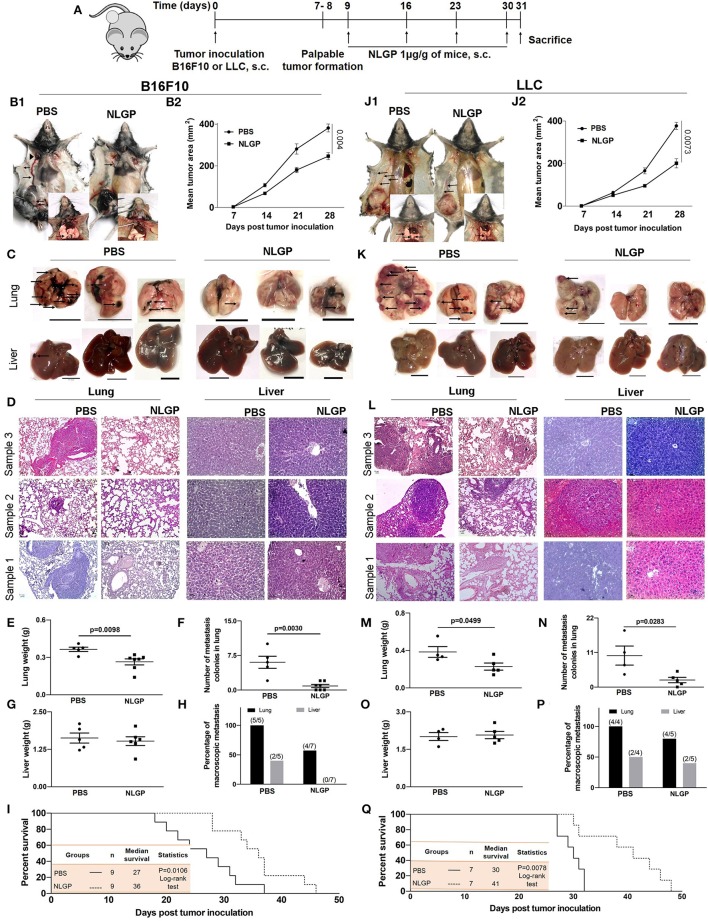
Neem leaf glycoprotein attenuates both the primary tumor and spontaneous metastasis of B16F10 and LLC. **(A)** Experimental design showing tumor inoculation and NLGP treatment. The tumor was inoculated subcutaneously (s.c.) (*n* = 3, repeat for three times for B16F10 and *n* = 3, repeat for two times for LLC), and after the formation of palpable tumor, mice were treated with NLGP once a week for 4 weeks. **(B1,J1)** Representative photographs of primary tumor on day 31 after s.c. inoculation of B16F10 and LLC in PBS- and NLGP-treated mice, respectively. Inset: Pulmonary metastasis of corresponding mice. Metastatic nodules are shown by arrows. Arrows indicate profound angiogenesis at ventral skin in the PBS group. Arrowhead indicates the node metastasis. **(B2,J2)** The tumor growth curves of B16F10 and LLC tumors in C57BL/6 mice. Each data point indicates mean tumor volume ± SEM. Representative photographs of lungs and livers harvested on day 31 post- **(C)** B16F10 and **(K)** LLC s.c. inoculation. Arrows point to surface metastatic nodules, which indicate 10 mm. Scale bar was assigned with the aid of ImageJ software after adjusting the scale with the original ruler. Both organs and ruler were photographed in the same frame. Representative hematoxylin-eosin staining of 5 μm lung (10×) and liver (20×) sections of B16F10 **(D)** and LLC **(L)** from PBS- and NLGP-treated mice. Arrow indicates metastatic foci. Scale bar 50 μ. Dot plots indicate **(E,M)** mean weight ± SEM of the lung; **(F,N)** numbers of metastatic nodules ± SEM; **(G,O)** mean weight ± SEM of the liver of mice harvested 31 days after s.c. B16F10 and LLC cell inoculation, respectively, in each case. Unpaired *t*-test was used to determine statistical significance. *P*-values are indicated in the corresponding graph. **(H,P)** Bar diagrams indicate the percentage of macroscopic metastasis in lung and liver from B16F10 and LLC inoculated. **(I,Q)** Kaplan-Meier survival analysis for B16F10 and LLC, respectively (*n* = 9 and *n* = 7 for B16F10 and LLC, respectively, repeat once). Data were analyzed by log-range test. Inset: Indicates mice number, median survival, and statistical value.

The antimetastatic potential of NLGP was evaluated in B16F10 spontaneous metastasis model (SMM) in C57BL/6J mice keeping PBS-treated mice as control (Figure 1A). In addition to the NLGP-mediated reduction in primary tumor (35.39%) (Figures 1B1,B2), a reduced number of metastatic nodules were observed on lung surfaces [Figure 1B (inset), Figures 1C,F]. Neem leaf glycoprotein therapy caused proportionally less increment in lung weight and size (due to the metastatic burden, Figure 1E). Histopathological analysis (Figure 1D) confirmed such notion, where we found few macroscopic metastases in the NLGP-treated group (Figure 1H) compared to the PBS control. In addition to lungs, NLGP therapy also reduced metastasis in liver (Figures 1C,D,G), spleen, kidney, and lymph nodes (Figures S1A,B), whereas no metastasis reduction was found in tumor-draining lymph nodes (data not shown). Kaplan–Meier survival analysis suggested increased mice survival in the NLGP-treated group [median survival increased from 27 to 36 days after NLGP treatment (Figure 1I)]. To validate the antimetastatic role of NLGP, we repeated identical experiment with LLC (ectopic model)–bearing mice, and similar trend was obtained (Figures 1J–Q), which delineates the wide application of NLGP in reducing primary tumor with different etiopathologies and their metastases.

### NLGP-Attenuated Metastasis Is Independent of the Primary Tumor Growth

**Figure 2:**
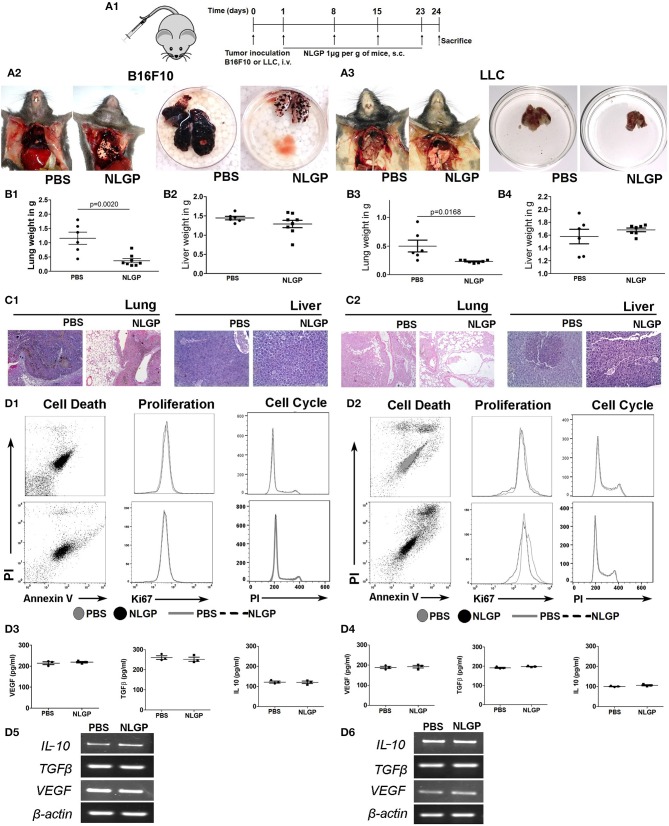
Neem leaf glycoprotein does not alter primary tumor cell properties. **(A1)** Schematic presentation of experiment design. Tumor cells were injected through t.v. followed by NLGP administration on the next day for once a week for 4 weeks, and mice were sacrificed following treatment completion (*n* = 3, for three times). **(A2,A3)** Representative photograph shows PBS- and NLGP-treated mice with its corresponding metastatic lungs (B16F10 and LLC), respectively. Dot plots indicate **(B1,B3)** mean lung weight ± SEM; **(B2,B4)** mean liver weight ± SEM of B16F10 and LLC bearing tumor host. **(C1,C2)** Representative hematoxylin-eosin–stained lung and liver sections of B16F10 and LLC mice. **(D1,D2)** B16F10 and LLC cells were treated with NLGP (1.5 μg/mL). Representative flow-cytometric dot plot overlaid, and histogram data represent the annexin V^+^ PI^+^, Ki67^+^, and PI^+^ cells from PBS- and NLGP-treated B16F10 and LLC cells (*n* = 1, repeat for three times). Neem leaf glycoprotein–treated and PBS tumor cell supernatant were analyzed for different cytokines by ELISA. Dot plots indicate mean amount of VEGF, TGF-β, and IL-2 for B16F10 **(D3)** and LLC **(D4)** cells, respectively (*n* = 1, repeat for three times; see Figures S2B.1,B.2; for statistical analysis). **(D5,D6)** Total RNA was isolated from NLGP- and PBS-treated tumor cells, and gene expression of *VEGF, TGF*β, and *IL-10* was analyzed against *beta-actin* as housekeeping gene (*n* = 1, repeat for three times).

Considering the linear relationship between primary tumor volume and metastasis, we checked the impact of NLGP-mediated primary tumor reduction on metastasis development. Accordingly, we used the experimental metastasis model (EMM) where tumor cells were inoculated intravenously without developing any primary tumor. On treatment completion (Figure 2A), we found a marked decrease in metastatic foci on the lung surface in NLGP cohort (Figures 2A2,A3) than PBS-treated mice in both melanoma and carcinoma models. Organ weight (Figures 2B1–B4) and histopathology (Figures 2C1,C2) data collectively support this notion. Neem leaf glycoprotein treatment also reduced distant metastasis in other organs (Figure S2A) with increased mice survival (Figures S2B.1,B.2).

As intrinsic and/or extrinsic properties of tumor cells influence metastasis, we assessed the effect of NLGP on relevant biological parameters, for example, apoptosis, proliferation, cell cycle, and cytokine production of B16F10 and LLC cells. Data obtained from these assays confirmed that *in vitro* NLGP treatment has no direct effect on tumor cells or their secretomes (Figures 2D1–D6; Figures S2C.1–C.4). Cumulative data suggest primary tumor reduction and/or alteration of basic primary tumor cell properties are not responsible for the NLGP-mediated metastasis inhibition. The possibility of NLGP-mediated alteration on EMT and invasion is also ruled out, as these steps are absent in EMM.

### NLGP-Attenuated Metastasis Is Independent of Tumor Cell Migration

**Figure 3:**
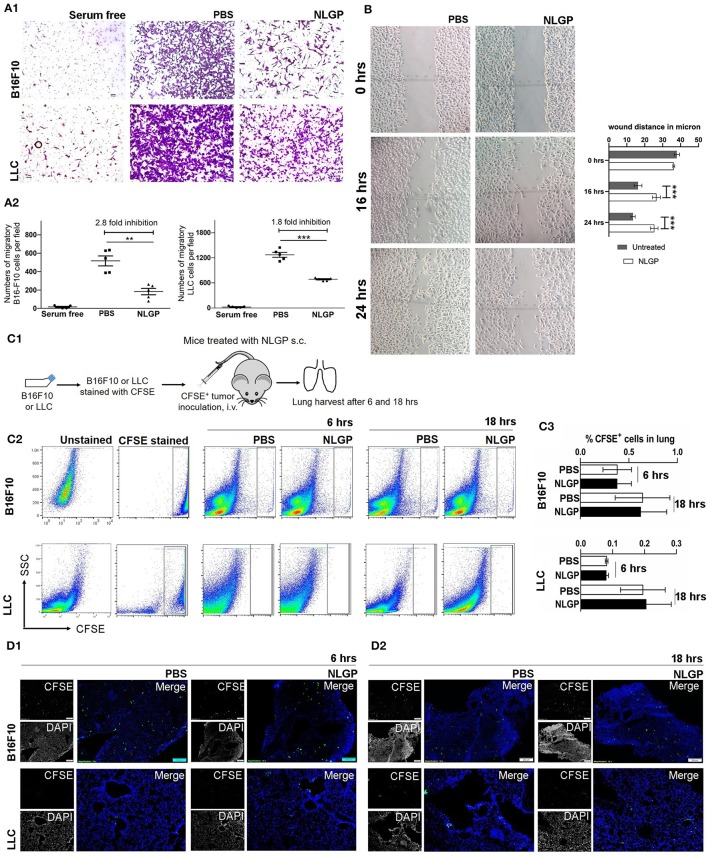
Neem leaf glycoprotein does not attenuate tumor cell migration. **(A1)** *In vitro* migration assay was performed in 8-μm pore Transwell insert with overnight serum starve tumor cells (B16F10 and LLC) against 10% heat-inactivated FBS in presence and absence of NLGP (1.5 μg/mL). Representative photographs of one of three independent experiments show crystal violet (0.01%)–stained migrated cells (after 16 h). Serum-free gradient was used as negative control. Dot plot **(A2)** indicates the mean number of migrated cells ± SEM of five separate fields from the same experiment. One-way analysis of variance (ANOVA) was used for statistical analysis followed by Tukey multiple-comparisons test. **(B)** A scratch wound was made in a full confluent B16F10 cell layer with a cell scratcher. Wound healing was monitored in a time-dependent manner in presence or absence of NLGP (1.5 μg/mL). Representative photograph shows one of three independent experiments. Wound closure was measured by using ImageJ by calculating the distance between the edge manually. Two-way ANOVA was used for statistical analysis followed by Bonferroni test. ***p < 0.001. **(C1)** Schematic experimental design. Tumor cells (B16F10 and LLC) were stained with CFSE followed by t.v. injection (*n* = 3, repeats for one). Mice were sacrificed following 6 and 18 h of tumor inoculation, and lungs were harvested. **(C2)** Representative dot plot image of flow-cytometric analysis of CFSE^+^ tumor cells in lungs after 6 and 18 h of tumor inoculation. **(C3)** Bar diagram indicates mean CFSE^+^ tumor cells ± SEM (*n* = 3). **(D1,D2)** Representative immune-fluorescence image of harvested lung sections at 10× magnification (*n* = 3, repeat for one). CFSE^+^ tumor cells are indicated by green color. Scale bar 200 μm. **p < 0.005.

As we know the antimetastasis effect of NLGP is not dependent on the reduction of the primary tumor growth, next, we hypothesized that NLGP may alter cancer cell migration to prevent metastasis. Results obtained from *in vitro* Transwell migration (Figures 3A1,A2) and scratch wound healing assay (Figure 3B) revealed that NLGP inhibits migration. To validate these *in vitro* results *in vivo*, CFSE-labeled tumor cells (B16F10 or LLC) were injected t.v. (Figure 3C1), and lungs were harvested from NLGP-treated and control mice following 6 and 18 h. However, no significant difference in the accumulation of CFSE^+^ tumor cells in the lungs from both groups of mice was observed by flow cytometry (Figures 3C2,C3) and immunofluorescence analysis (Figure 3D1; Figure S3A). The discrepancy between *in vitro* and *in vivo* observations suggests that *in vivo* NLGP treatment did not attenuate tumor cell migration to lungs to prevent metastasis. We further assume that direct interaction of NLGP with tumor cells *in vitro* might result to this attenuation, and such “direct interaction” was absent *in vivo*. To validate this, *in vitro* NLGP–pretreated (for 24 h) tumor cells were inoculated t.v., and significantly fewer number of tumor cells were detected in the lungs compared to the PBS-treated group (Figures S3B.1–B.5), supporting our earlier assumption.

On the other hand, an equal number of CFSE^+^ tumor cells were detected in lungs from both groups after 18 h, suggesting NLGP treatment did not affect the extravasation and initial survival of tumor cells in lungs (Figures 3C2,D2).

Of note, *in vitro* NLGP treatment also reduced invasion (Figures S3C.1,C.2) and tube formation in B16F10 tumor cells (Figure S3D).

### NLGP Augments Immune-Mediated Killing and Prevents Systemic Neoangiogenesis to Hamper Colonization of Extravagated Cells

**Figure 4:**
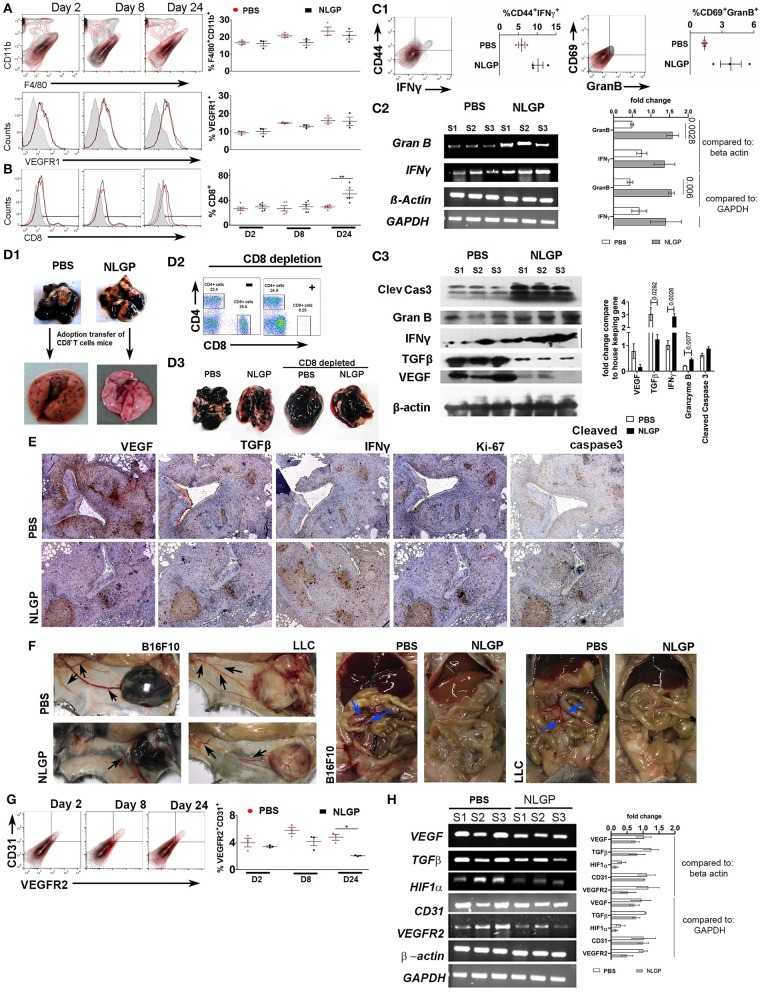
Neem leaf glycoprotein intervenes metastatic colonization. Representative overlaid counter plot and histograms show the status of **(A)** F4/80^+^CD11b^+^ and VEGFR1^+^ and **(B)** CD8^+^ T cells. Corresponding dot plot indicates mean percentage of target cells ± SEM (*n* = 3, repeat for one, for F4/80^+^CD11b^+^ and VEGFR1^+^, and *n* = 5 for CD8^+^ T cells repeat for one). **(C1)** Representative figure indicates presence of CD44^+^IFN-γ^+^ and CD69^+^GranB^+^ cells within gated CD8^+^ T cells on day 24 in PBS- and NLGP-treated lungs. Corresponding dot plot indicates percentage of target cells ± SEM (*n* = 3, repeat for one). **(C2)** Different gene transcription was analyzed by RT-PCR. Agarose gel shows the expression of *IFN*γ and *GranB* transcripts. Corresponding bar diagram indicates the fold change in expression as measured by ImageJ software. **(C3)** Total protein was isolated from PBS- and NLGP-treated lungs (day 24; *n* = 3, repeat for one) to check the expression of cleaved caspase 3, granzyme B, IFN-γ, TGF-β, and VEGF at protein level by Western blot. Expression intensity was measured by LI-CORE lite software. Unpaired *t*-test was performed to analyze statistical significance. The *p*-values are indicated in figure. **(D1)** CD8^+^ T cells were isolated by magnetic bead positive selection from PBS- and NLGP-treated mouse lung (day 24) and adoptively transferred into another group of mice with metastases (see also Figure S4DA). The second group of mice was sacrificed, and lungs were harvested. Representative lung image indicates fewer number of metastatic nodules in mice, received adoptively transferred CD8^+^ T cells from NLGP-treated mice (*n* = 3, repeat for one; see also Figures S4DA.1,A.2). **(D2)** Flow-cytometric dot plot shows the status of CD8^+^ T cells in peripheral blood after *in vivo* CD8^+^ T cell depletion (for details, see Figure S4DB.1). **(D3)** Representative photograph of lungs from different groups shows that CD8^+^ T cell depletion majorly abrogate NLGP's antimetastatic efficiency (*n* = 3, repeat for one and Figure S4FB.2). **(E)** Immunohistochemistry was performed in paraffin-embedded sequential lung sections (5 μm) for VEGF, TGF-β, IFN-γ, Ki-67, and cleaved caspase 3 from PBS- and NLGP-treated mice. **(F)** Representative image shows tumor (B16F10 and LLC)–induced angiogenesis in PBS- and NLGP-treated mice for both SMM and EMM. Black arrows indicate angiogenesis at ventral skin. Blue arrows indicate angiogenesis at mesenteric site (see Figures 1B1,J1; Figures S4FA,B). **(G)** Representative flow-cytometric overlaid counter plot indicates presence of CD31^+^ and VEGFR2^+^ cells in PBS- and NLGP-treated mice lungs (day 24, *n* = 3, repeat for one). Corresponding dot plot indicates percentage of target cells ± SEM (*n* = 3). **(H)** Agarose gel electrophoresis data show the expression of *VEGF, TGF*β, *HIF1*α, *CD31*, and *VEGFR2* transcripts in three different samples against the housekeeping gene *beta-actin* and *GAPDH*. (N.B., **C2,H** used the same housekeeping gene as control; as both are analyzed from same lung samples) Corresponding bar diagram indicates the fold change in expression. Expression intensity was measured by ImageJ software. *p < 0.05, **p < 0.005.

As migration and extravasation of metastatic cells remain unaltered after NLGP treatment, next, we checked NLGP's effect on colonization. Colonization (2, 7) depends on the premetastatic niche (18) formation with a simultaneous escape from immune-mediated killing (19–21) and induction of neoangiogenesis (22, 23). Hence, we measured the presence of CD11b^+^F4/80^+^, VEGFR1^+^ (24) (Figure 4A), and other related cells, which aid in niche formation (Figure S4AA), within lungs at the early time point (day 2 after tumor inoculation). Presence of identical number of cells in both groups indirectly indicates that NLGP did not alter the metastatic niche formation to abrogate metastasis.

To elucidate the NLGP's effect on immune-mediated tumor cell killing, first, we checked the CD8^+^ T cell activation status in EMM and found elevated proportion of splenic CD8^+^, CD8^+^Ki67^+^, CD8^+^CD44^+^, CD8^+^IL-2^+^, and CD8^+^IFN-γ^+^ T cells in NLGP-treated cohort (Figures S4BA,B), which led us to hypothesize that activated CD8^+^ T cells might infiltrate into the lungs to reduce metastasis. High lung infiltration of CD8^+^ T cells (Figure 4B) further confirmed these observation. Lung infiltrated CD8^+^ T cells were also CD44^+^IFN-γ^+^ and GranB^+^CD69^+^ (Figure 4C1; Figure S4CA). In order to assess the cytotoxic potential of these CD8^+^ T cells, *ex vivo* LDH release assay was performed, and high cytotoxic ability of CD8^+^ T cells was documented toward B16F10 cells. Antigen restimulation (with B16F10 antigen) assay revealed higher IFN-γ release from the same T cells of the NLGP-treated mice group only (Figures S4CB,C) in both cases. To further confirm the functionality of these CD8^+^ T cells *in vivo*, we measured the expression of IFN-γ, granzyme B, and cleaved caspase 3 in lung microenvironment and detected higher expression of these molecules in NLGP-treated cohort (Figures 4C2,C3). Next, cleaved caspase 3 expression was measured to assess the death of tumor cells within lung nodules, which support greater tumor cell killing in the NLGP cohort (Figure 4E). Assessment of proliferation status using Ki67 marker showed lesser proliferation of tumor cells in NLGP-treated cohort (Figure 4E). No major alteration was found in CD5^+^CD19^+^, NK1.1^+^IFN-γ^+^, and CD11c^+^CD11b^+^ cells (day 24, Figure S4AA).

To further corroborate the contribution of CD8^+^ T cells in NLGP-mediated metastasis prevention, we isolated CD8^+^ T cells from metastatic lungs of NLGP and PBS-treated mice and adoptively transferred these CD8^+^T cells (2 × 10^5^) into a separate mice cohort, already inoculated with B16F10 cells (72 h earlier via t.v. injection; Figure S4DA). After 15 days, mice with adoptively transferred CD8^+^ T cells from the NLGP cohort exhibited reduced pulmonary metastasis in comparison to the PBS-treated group (Figure 4D1; Figures S4D,A.2,A.3). In a separate experiment, we systematically and specifically depleted CD8^+^ T cells *in vivo* using specific depleting antibody 24 h before NLGP injection (Figure S4DB.1) and found that CD8^+^ T cell depletion eliminates NLGP's effect on metastasis (Figures 4D2,D3; Figure S4DB.2). Accordingly, low expression of cleaved caspase 3 was observed after CD8^+^ T cell depletion in lungs of the NLGP-treated group Figure S4DB.3). Furthermore, treatment with *ex vivo* NLGP-treated CD8^+^ T cell does not reduce metastasis compared to *ex vivo* PBS-treated CD8^+^ T cell treatment (Figures S4EA1–A3). Based on all these results, we concluded that NLGP treatment orchestrated CD8^+^ T cell–mediated tumor cell killing to reduce colonization, thereby metastasis.

Furthermore, we macroscopically found a reduction in angiogenesis in the NLGP-treated mice from SMM and EMM (Figures 1B1,J1, 4F; Figures S4FA,B). Assessment of the status of angiogenesis revealed a smaller number of CD31^+^VEGFR2^+^ endothelial cells in lungs from mice of the NLGP cohort, indicating lesser angiogenesis (Figure 4G). Simultaneously, VEGF, TGF-β, and hypoxia-inducible factor 1α (HIF-1α) expression was downregulated in lungs (Figures 4E, H) of mice from the NLGP cohort. Evans blue injection shows reduced angiogenesis in the NLGP-treated group with documentation of less leakiness in blood vessels (Figure S4G).

### NLGP Modulates DC Functions to Activate CD8^+^ T Cells

**Figure 5:**
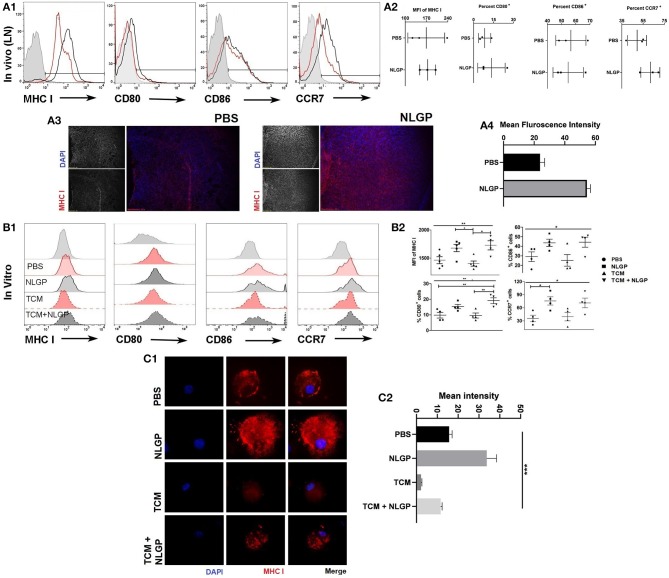
Neem leaf glycoprotein–activated DCs modulate CD8^+^ T cells. **(A1)** Following 48 h of NLGP administration, TDLN was isolated, and MHC-I, CD80, CD86, and CCR7 expressions were measured by flow cytometry (*n* = 3, repeat for once). **(A2)** Bar diagram indicates the mean of MFI ± SEM of MHC-I or mean percentage positive ± SEM of CD80, CD86, and CCR7 (*n* = 3, repeat for once). **(A3)** Representative image shows the MHC-I expression in PBS- and NLGP-treated TDLN (*n* = 3, repeat for one). **(A4)** Bar diagram indicates mean fluorescence intensity of MHC-I expression ± SEM of lymph node. **(B1)** Immature BmDCs (day 6) were treated with and without TCM and NLGP for 32 h followed by analysis of MHC-I, CD80, CD86, and CCR7 expression. Representative offset histograms show expression of MHC-I, CD80, CD86, and CCR7 (*n* = 1, repeat for five times for MHC-I and four times for CD80, CD86, and CCR7). **(B2)** Dot plot shows the mean target cells ± SEM. One-way ANOVA was used followed by Tukey multiple comparisons. **(C1)** Representative image shows the surface MHC-I expression on BmDCs. **(C2)** Bar diagram represents mean MHC-I expression intensity ± SEM (*n* = 1, repeat for three times). *p < 0.05, **p < 0.005.

Considering the central role of CD8^+^ T cells for NLGP-influenced antimetastasis, next, we studied the mechanism of CD8^+^ T cell activation during NLGP therapy. As naive T cell activation requires support from professional APCs, mainly from DCs (25), we studied *in vivo* modulation of DCs by NLGP. Tumor-draining lymph node (TDLN) cells were collected after 48 h of NLGP treatment and found higher per-cell MHC-I expression along with increased CD80 and CCR7 in comparison to control (Figures 5A1,A2, also in contralateral lymph node and tumor; Figure S5AA). Immunofluorescence analysis of MHC-I in TDLN also supports this notion (Figures 5A3,A4). For further validation, BmDCs Figure S5BA; optimum upregulation in 32 h; Figure S5BB) were exposed to tumor-conditioning media (TCM) to mimic tumor milieu, and upregulated MHC-I, CD80, and CCR7 expression was observed (Figures 5B1,B2) following NLGP supplementation (CD11c^+^ cells 77.77–89.93%; Figure S5DC). MHC-II was also upregulated in this condition by NLGP (Figures S5CA,B). NLGP induced upregulation of MHC I also evident by immunofluorescence (Figures 5C.1,C.2).

In addition to TDLN-DCs, next, we checked MHC-I expression in lung CD103^+^ DCs; however, no NLGP-mediated alteration was noted (Figure S4AA). This result suggests that NLGP might not directly modulate lung DCs to activate T cells; rather, activated T cells possibly migrate from TDLNs. To check this notion, we analyzed some candidate lung homing markers such as CXCR3, CCR4, CCR5, and CCR6 (26–28) in lung infiltrated CD8^+^ T cells, and higher proportion of CXCR3^+^CD8^+^ and CCR4^+^CD8^+^ T cells was detected (Figure S5DA) in the NLGP-treated cohort. However, expression of CCL22, CCL17 and CXCL10, CXCL9 [chemoattractant for CCR4 and CXCR3, respectively (28)] revealed no significant difference in transcript levels (Figure S5DB). These data indicate that the higher infiltration of CXCR3^+^CD8^+^ and CCR4^+^CD8^+^ T cells in lung of the NLGP-treated cohort was due to the greater abundance of CXCR3^+^CD8^+^ and CCR4^+^CD8^+^ T cells. Next, we validated this *in vivo* observation *in vitro*; TCM-exposed BmDCs imprinted the same homing marker (CXCR3 and CCR4) on purified CD8^+^ T cells after DC:T (1:10) cell coculture (Figure S5DC) in presence of NLGP.

### NLGP-Mediated MHC-I Upregulation Is Translation Dependent, but Transcription Independent

**Figure 6:**
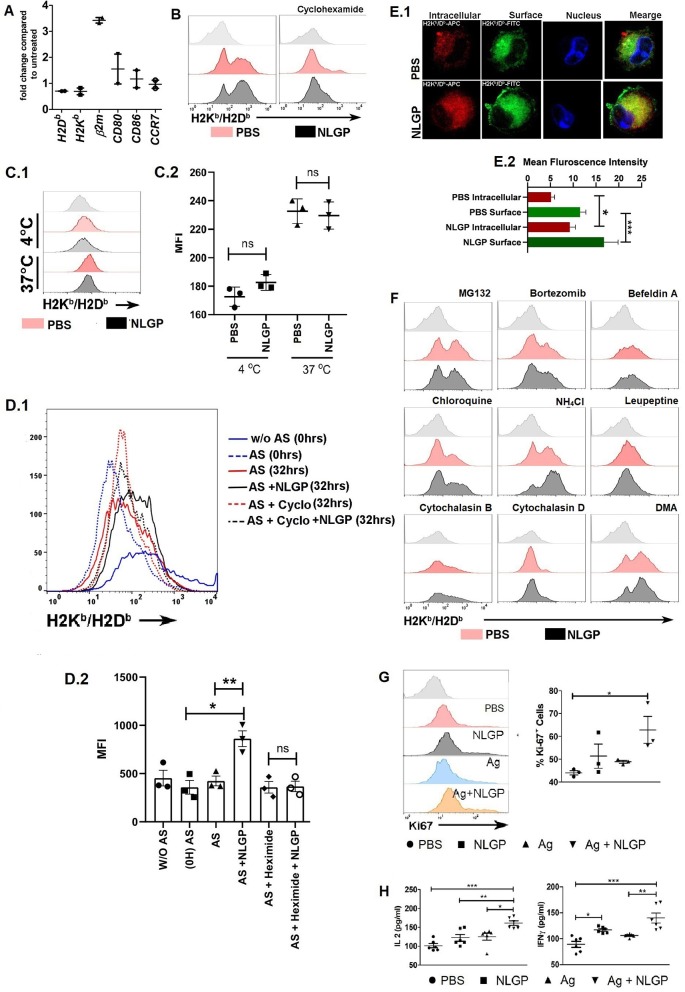
Neem leaf glycoprotein–mediated MHC-I upregulation is translation dependent. **(A)** Expressions of *H2K, H2D*, β*2m, CD80, CD86*, and *CCR7* were assessed by real-time PCR. Data were analyzed by delta-delta-Cq methods compared to β*-actin* (*n* = 1, repeat for once) (*n* = 2). **(B)** Effect of cycloheximide (5 μM) H on NLGP-induced MHC-I upregulation was assayed. Offset histogram plot represents one of the experimental data of three independent experiments (*n* = 1, repeat for three times; see also Figure S6DA). **(C1)** Effect of NLGP on normal MHC-I endocytosis was determined. Representative histogram figure shows NLGP does not alter MHC-I endocytosis. **(C2)** Dot plot indicates mean MFI ± SEM of MHC-I molecules from MHC-I endocytosis assay. Data indicate that at 37°C NLGP did not alter MHC-I endocytosis (*n* = 1, repeat for three times). Data were analyzed by one-way ANOVA. **(D1)** Representative data show one result from three independent experiments of acid striping. **(D2)** Dot plot indicates mean MFI ± SEM of MHC-I molecules from acid stripping experiment (*n* = 1, repeat for three times). Data were analyzed by one-way ANOVA. **(E1)** Representative image shows the expression of intracellular and surface MHC-I by confocal microscopy (*n* = 1, repeat for four times). **(E2)** Bar diagram represents the mean intensity ± SEM of intracellular and surface MHC-I. **(F)** Effect of different inhibitors on the NLGP-induced MHC-I upregulation (see also Figure S6DA) **(G)** Representative histogram represents expressions of Ki-67. Dot plot shows the mean ± SEM (*n* = 1, repeat for three times). **(H)** Dot plot indicates mean IL-2/IFN-γ ± SEM by ELISA (*n* = 2, repeat for three times). One-way ANOVA was used and followed by Tukey multiple comparisons. *p < 0.05, **p < 0.005, ***p < 0.0005.

Interestingly, NLGP-mediated MHC-I upregulation was transcription independent; as we found, there was no alteration in the mRNA transcript level of genes such as H2K, H2D, CD80, CD86, and CCR7 by real-time PCR (Figure 6A). *Tapasine, TAP1, TAP2, PA28*α, *PA28*β, *PA28*γ, *LMP2, LMP7, LMP10, ERp57, calnexin, calreticulin, cathepsin S, cathepsin B, cathepsin L, cathepsin D*, and *CIITA* genes were also analyzed by semiquantitative RT-PCR (Figures S6AA.1,A.2). But treatment with cycloheximide (protein translation inhibitor) partially hampers NLGP-mediated MHC-I upregulation (Figure 6B; Figure S6DA), indicating NLGP-mediated MHC-I alteration is translation dependent. As cycloheximide inhibits translational machinery (29), we were unable to specify any protein(s), getting altered after NLGP treatment. To explain NLGP-mediated MHC-I translation, we measured first whether there is any alteration in MHC-I endocytosis in the presence of NLGP, because reduction in endocytosis may give rise to higher surface MHC-I expression. Neem leaf glycoprotein did not alter MHC-I endocytosis (Figures 6C1,C2), indicating that NLGP-mediated MHC-I upregulation is not due to alteration in endocytosis. Next, we wanted to check whether the NLGP-induced increment of MHC-I occurs due to export of antigen-loaded MHC-I from the cytosol rather than retention of MHC-I molecule on surface over time. We stripped all the surface MHC-I by acid stripping, followed by NLGP treatment. The NLGP-treated group exhibits more surface MHC-I expression compared to control (Figures 6D1,D2), confirming the NLGP-mediated export of MHC-I from the cytosol to surface. Accordingly, enhanced surface expression of MHC-I was noted. To further verify the enhancement, we treated the BmDCs with cycloheximide after acid stripping, and the NLGP-induced MHC-I increment was hampered (Figures 6D1,D2) in presence of cycloheximide. Next, we checked whether there is a reduction in intracellular pool of MHC-I during NLGP-mediated increment of surface MHC-I. To address this, we stained surface and intracellular MHC-I by differently tagged anti-H2K^b^/H2D^b^ antibody and assessed using flow cytometry, immunofluorescence (Figures S6BA,CA), and confocal microscopy (Figures 6E.1,E.2). Such analysis revealed that NLGP treatment increases both the surface and intracellular MHC-I, suggesting the potentiation of MHC-I translation by NLGP.

As NLGP upregulates MHC-I, next we wanted to know how alterations in antigen processing and presentation affect this upregulation. We found that in the presence of proteasome inhibitors, MG132 and bortezomib, NLGP-mediated MHC-I upregulation was inhibited. This observation clearly suggested that, in the absence of proteosome-generated peptide, NLGP treatment does not increase the surface expression of MHC-I and hence does not alter the basic principle, which states that only antigen-peptide–bound MHC-I gets exported to the cell surface. Protein transport inhibitor, brefeldin A, also inhibits NLGP-mediated MHC-I upregulation, which is also in line with the basic principle of cellular export, whereas phagosome and lysosome fusion inhibitor (chloroquine and NH_4_Cl) enhanced NLGP-induced MHC-I expression. As phagosome and lysosome fusion gets inhibited by chloroquine and NH_4_Cl, it creates opportunity for antigen within phagosome to get export into cytosol for proteasome-mediated degradation. Hence, this leads to increase in total pool of proteasome-derived peptide, which in turn augments in the NLGP-mediated MHC-I increment. Cysteine protease inhibitor, leupeptin, which inhibits the cathepsin family protein within the phagolysosome, probably does not aid in antigenic protein export within cytosol, and hence does not augment NLGP-mediated MHC-I upregulation to a significant level. Phagocytosis inhibitors, cytochalasin D and cytochalasin B, and macropinocytosis inhibitor, dimethyl amiloride, did not show any effect (Figure 6F; Figures S6DA,B). From these results, we concluded that NLGP-mediated MHC-I upregulation is proteasome sensitive, and increase in cytosolic protein (by treatment with chloroquine, NH_4_Cl) augments this upregulation and indicates the possible involvement of NLGP in cross-presentation as cross-presentation is very relevant in tumor biology, where extracellular proteins are presented in the context of MHC-I.

Next, we addressed whether the presence of antigen (30) can induce the synergistic effect with NLGP, but detected no synergistic effect for both soluble tumor cellular lysate and necrotic antigen (Figures S6EA,B) in respect to MHC-I expression, but an upsurge of CD8^+^ T cell proliferation (Figure 6G) with IL-2 and IFN-γ production (Figure 6H) was found in antigen + NLGP group compared to the NLGP group when these DCs were cocultured with CD8^+^ T cells. To investigate further, we used OVA and OVA_257−264_ (SIINFEKL) as a foreign antigen. Two different antibodies were used; one specifically detects SIINFEKL-bound H2K^b^, and the other detects antigen-bound H2K^b^/H2D^b^. More MHC-I expression was detected by H2K^b^/H2D^b^-specific antibody in the NLGP-treated group, whereas slight increment was detected by SIINFEKL-specific H2K^b^ (Figures S6FA,B). Hence, we concluded that NLGP indeed aids to export peptide-bound MHC-I [both SIINFEKL-bound (generate from OVA or SIINFEKL peptide) and cytosolic peptide–bound] molecules independent of their nature.

## Discussion

In context to the tumor growth restriction by NLGP (11–15), here, we explored the antimetastatic role of NLGP using preclinical models, SMM (2, 7, 31) and EMM (31, 32). Metastatic growth is still uncontrollable by modern therapy (8–10). Thus, a new regimen to control metastasis is highly desired. Along with reported tumor growth restricting property of NLGP, it also reduces the associated metastasis, as seen in B16F10 and LLC models. Interestingly, the antimetastatic property of NLGP does not depend on alteration in primary tumor, as we observed metastasis inhibition in EMM (no primary tumor) also. This observation established NLGP as a better choice of drug in postsurgical occult metastasis treatment, because in the absence of primary tumor, NLGP is effective to reduce metastasis. Moreover, in the light of “parallel progression model” (33), contrary to “linear progression model” (2), it becomes evident that initiating an early treatment with antimetastatic therapy might be more beneficiary to manage the micrometastasis, which is generally present during initial detection of the primary tumor. Henceforth, anticancer therapy with NLGP will give an edge over the other drugs targeting primary tumors only.

Mechanistically, NLGP does not interfere with intrinsic tumor cell properties such as cellular proliferation, cell cycle, apoptosis, and cytokine secretion and steps such as migration, extravasation, and premetastatic niche formation (24). In fact, niche formation concept was first coined by Stephen Paget in his “seed and soil” hypothesis (31) in 1889. Recently, with the advent in knowledge on tumor microenvironment and stromal cell biology, many researchers are interested to target the niche or soil (34, 35) instead of metastatic cells for its prevention. Despite no significant alteration in metastatic soil, NLGP intervenes the last and rate-limiting step of invasion metastasis cascade known as colonization (2, 36, 37). Colonization or formation of macrometastasis is strictly controlled by the balance between proliferation and elimination of tumor cells, which is under immune surveillance (19–21). Moreover, induction of neoangiogenesis (22, 23) promotes colonization. Our initial macroscopic and microscopic (histopathological) analyses have clearly shown a reduced number of the metastatic colony in different organs of the NLGP-treated mice. Both reduced size and colony number indicate attenuation of colonization. Mechanistically, apart from the systemic induction of type 1 immune activation, a greater number of CD8^+^ T cells were detected in metastatic lungs from NLGP-treated mice to prevent colonization. The principal limitation for any T cell–based therapy is functional exhaustion of tumor-reactive T cells (38, 39), and such exhaustion limits the success of several immunotherapeutics (18). Interestingly, NLGP-treated T cells from tumor hosts are in activated state, as discussed in detail in the *Results* section. *In situ* examination of cleaved caspase 3, *ex vivo* LDH release assay, and antigen-restimulation assay suggest infiltrated CD8^+^ T cells are functionally active. We further confirmed the involvement of NLGP-activated CD8^+^ T cells in metastasis reduction, by a pair of reversible experiments, either by introducing NLGP-activated CD8^+^ T cells or by depleting CD8^+^ T cells. Adoptive transfer of NLGP-activated CD8^+^ T cells remarkably reduces the metastatic burden. On the other hand, CD8^+^ T cell depletion in the NLGP-treated mice evidenced no antimetastatic activity. Additionally, we found reduced atrophied thymus in NLGP-treated tumor hosts (Figure S7), probably aiding to maintain overall CD8^+^ T cell pool participating in metastasis reduction. In metastatic mice, atrophied thymus causes arrest in DN2 to DN3 transition, resulting from a scarcity of double-positive as well as single positive CD8^+^ T cells, which leads to metastasis promotion (Guha et al.; unpublished observation). Apart from CD8^+^ T cell–dependent killing, we macroscopically detected reduced angiogenesis in the tumor and its adjacent areas; those are which were in line with the previous observation (13). Reduced angiogenesis (low level of CD31^+^ and VEGFR2^+^ cells), along with the decreased level of angiogenesis regulatory factors such as VEGF and TGF-β, was detected in metastatic lungs after NLGP treatment. This may have an indirect role in metastasis attenuation. However, this study does not demonstrate the particular mechanism of reduced angiogenesis, but immune-mediated (mainly by IFN-γ) angiogenesis inhibition is already documented by us (13) and others (40).

However, NLGP does not activate CD8^+^ T cells directly; rather, NLGP modulates altered APC functions. In tumor condition, APCs such as DCs become immature and lose its primary antigen-presenting ability (41), which gets rectified by NLGP. Neem leaf glycoprotein enhances the surface expression of MHC-I, CD80, and CCR7 on DCs and simultaneously specifically imprints CCR4/CXCR3 homing receptor expression on T cells. This helps in regaining activation of naive CD8^+^ T cells with the homing capacity to the primary tumor (42) and multiple metastatic sites (28), although other homing receptors (28) are not studied here except CCR5 and CCR6 with showing expression.

We recently established that NLGP acts via dectin-1 receptor to modulate DCs (Ganguly et al.; unpublished observation), and further elucidation of the signaling pathway is ongoing. T cell activation by dectin-1–stimulated DCs has been reported by others in context to fungal infection; however, the literature on antitumor role of dectin-1–activated DCs is very limited (43–48). LeibundGut-Landmann et al. (43) only showed that dectin-1–activated DCs promote antimetastatic cytotoxic CD8^+^ T cells, whereas other studies focus on the primary tumor (44, 45). The uniqueness of NLGP relies on the fact that it aided the export of the peptide:MHC-I complex, depending on the quantity of self and/or antigenic peptidome (which are generated by proteasomal cleavage) present within cytosol rather than the quality of the peptidome. This feature ensures that NLGP-treated DCs give rise to functional T cell activation only in the presence of tumor-associated or tumor-specific antigen. Interestingly, a similar observation was also made by LiebundGut-Landmann et al., where CD8^+^ T cell proliferation only occurred by curdlan-induced dectin-1–activated DCs in the presence of antigen (43). Overall, the stringent requirement of antigen in CD8^+^ T cell activation by dectin-1–induced DC reduced the chances of bystander immunotoxicity, which occurs with many immunotherapeutic drugs (49, 50). On the other hand, this particular feature theoretically predicts that NLGP will be beneficial for a tumor with multiple antigens such as melanoma but may not be beneficiary against tumor with minimal tumor antigen.

Validation of NLGP's effect in preclinical human tumor models is not possible because of CD8^+^ T cell dependence of NLGP action. Preclinical exploration of human tumor model specifically demands the use of nude or SCID mice, and those are immune compromised and lack CD8^+^ T cells, in particular.

A conglomeration of all these results proves that NLGP, via altering maturation of DCs, generates antigen-specific activated CD8^+^ T cells, which skews the ongoing dialogue between the immune system and cancer cells to prevent the primary tumor growth and metastatic colonization. Based on our accumulated data from present or previous studies, we are in good faith that adjuvant and/or neoadjuvant treatment with NLGP in combination with other immunotherapy and/or chemotherapy will exhibit synergistic benefits in clinical outcome.

## Data Availability Statement

The data and material related to the findings of this study are presented in main figures within the article or as supplementary information files (**Additional Files**). Raw data related to this publication are available from the corresponding author on reasonable request.

## Ethics Statement

This study was approved by the Animal Ethical Committee of the Chittaranjan National Cancer Institute, Kolkata, India (Approval No. IAEC-1774/RB-1/2015/3).

## Author Contributions

ABh, ABo, and RB designed the study, analyzed the data, and wrote the manuscript. ABh, IG, and NG performed the research. AS and SD performed PCR and WB. AD performed confocal microscopy. PN, SG, TG, EH, and SB provide resources. ABo and RB supervise the project. RB acquired the fund. All authors read and approved the final manuscript.

### Conflict of Interest

The authors declare that the research was conducted in the absence of any commercial or financial relationships that could be construed as a potential conflict of interest.
