# Supplementary material for: NLGP Attenuates Murine Melanoma and Carcinoma Metastasis by Modulating Cytotoxic CD8+ T Cells
Source: Front Oncol. 2020 Mar 10;10:201. doi: 10.3389/fonc.2020.00201 (PMC7076076; doi:10.3389/fonc.2020.00201)
Supplement: Supplementary file 1 [file Data_Sheet_1.pdf]

NLGP attenuates murine melanoma and carcinoma metastasis by modulating cytotoxic CD8$^+$ T cells

Avishek Bhuniya[1], Ipsita Guha[1], Nilanjan Ganguly[1], Akata Saha[1†], Shayani Dasgupta[1†], Partha Nandi[1], Arnab Das[2], Sarbari Ghosh[1], Tithi Ghosh[1], , Enamul Haque[3], Saptak Banerjee, Anamika Bose[1], Rathindranath Baral[1]*

Additional File 1

Figure S1	Page 2
Figure S2	Page 3
Figure S3	Page 4
Figure S4A	Page 5
Figure S4B	Page6
Figure S4C	Page 7
Figure S4D	Page 8
Figure S4E	Page 9
Figure S4F	Page 10
Figure S4G	Page 11
Figure S5A	Page 12
Figure S5B	Page 13
Figure S5C	Page 14
Figure S5D	Page 15
Figure S6A	Page 16
Figure S6B	Page 17
Figure S6C	Page 18
Figure S6D	Page 19
Figure S6E	Page 20
Figure S6F	Page 21
Figure S7	Page 22
Figure Legend	Page 23
Table S1	Page 29

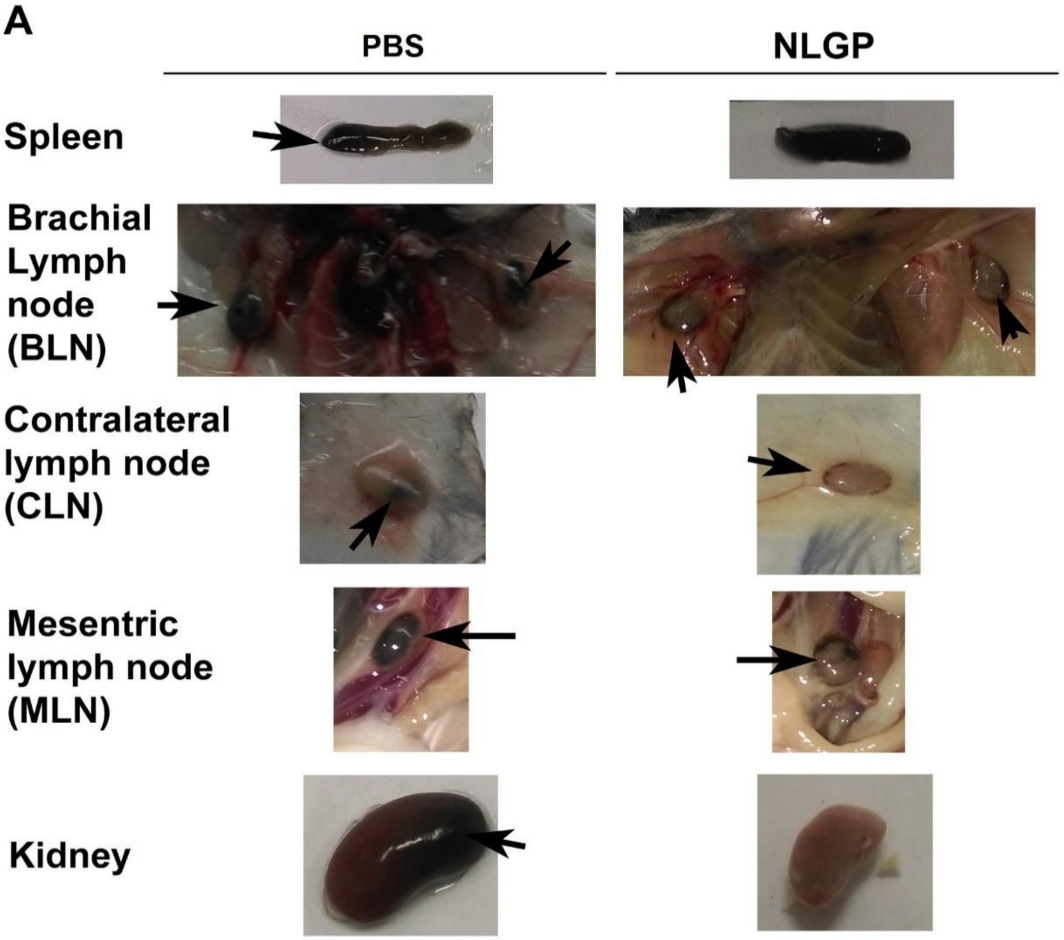

B

Number of mice along with percentage of macroscopic metastasis presented in parentheses

		Initial n	End point n	Lung	liver	Spleen	B LN	M LN	C LN	Kidney	Others
B16F10 Melanoma	PBS	9	5	**5** (100%)	**2** (40%)	**2** (40%)	**2** (40%)	**2** (40%)	**2** (40%)	**1** (20%)	1 (20%)
	NLGP	9	7	**4** (57%)	**0** (0%)	**0** (0%)	**1** (14%)	**1** (14%)	**1** (14%)	**0** (0%)	**0** (0%)
Lewis Lung Carcinoma	PBS	6	4	**4** (100%)	**0** (0%)	**0** (0%)	**0** (0%)	**0** (0%)	**0** (0%)	**0** (0%)	**0** (0%)
	NLGP	6	5	**4** (80%)	**0** (0%)	**0** (0%)	**0** (0%)	**0** (0%)	**0** (0%)	**0** (0%)	**0** (0%)

Figure S1

Bhuniya et al

A

		Initial No.	End point	Lung	Liver	Spleen	BLN	MLN	CLN	Kidney	Others
B16F10 Melanoma	PBS	9	6	6 (100%)	3 (50%)	2 (33%)	4 (66%)	3 (50%)	1 (16%)	2 (33%)	2 (33%)
	NLGP	9	8	6 (75%)	2 (25%)	0 (0%)	2 (25%)	1(12.5%)	0(0%)	1(12.5%)	0 (0%)
Lewis Lung Carcinoma	PBS	9	6	6 (100%)	4 (66%)	0 (0%)	0 (0%)	0 (0%)	0 (0%)	0 (0%)	0 (0%)
	NLGP	9	7	4 (57%)	2 (28%)	0 (0%)	0 (0%)	0 (0%)	0 (0%)	0 (0%)	0 (0%)

B.1

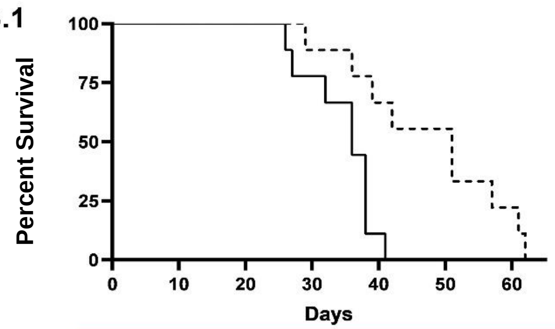

Group		n	median	Statistics
PBS	———	9	36	P=0.0038 Log-rank test
NLGP	---------	9	51	

B.2

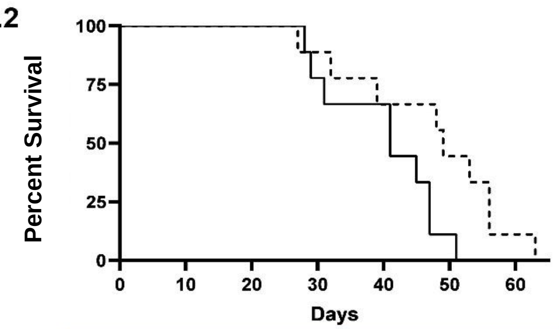

Group		n	median	Statistics
PBS	———	9	41	P=0.041 Log-rank test
NLGP	---------	9	49	

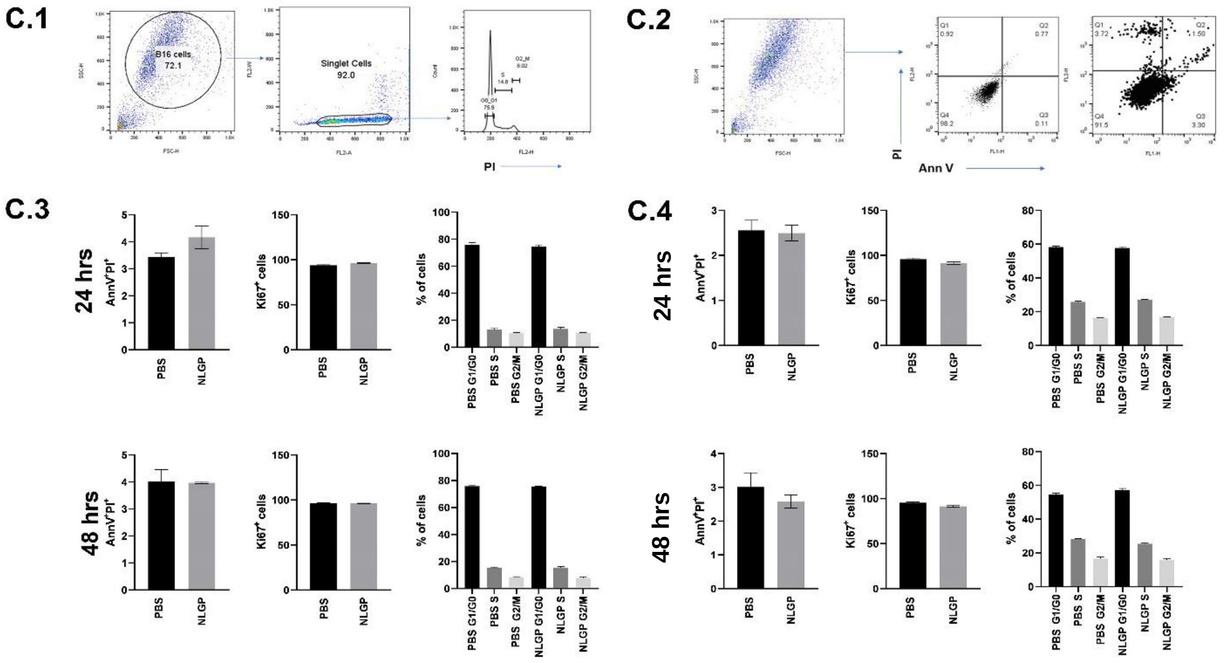

Figure S2

Bhuniya et al

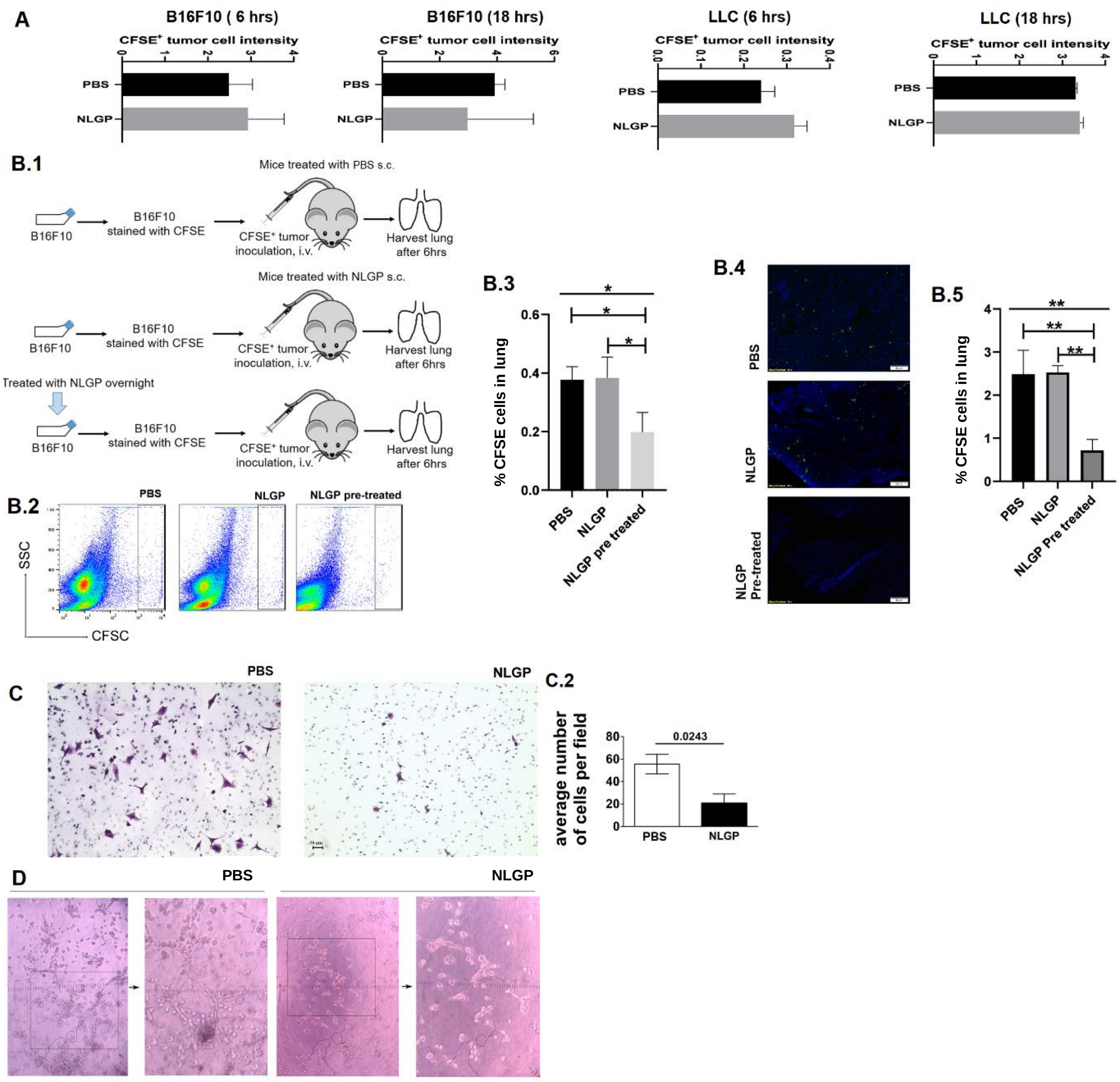

Figure S3

Bhuniya et al

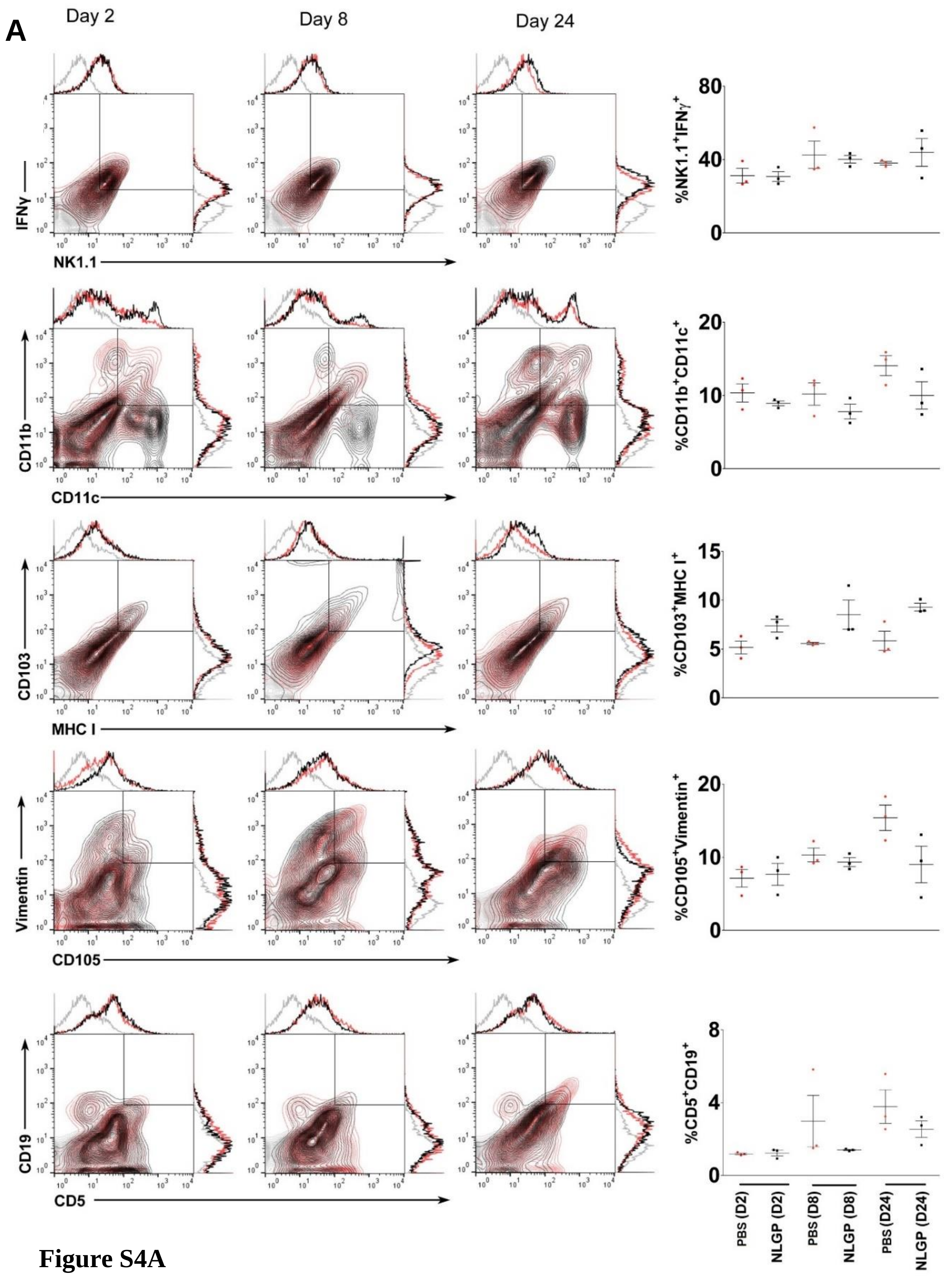

Figure S4A

Bhuniya et al

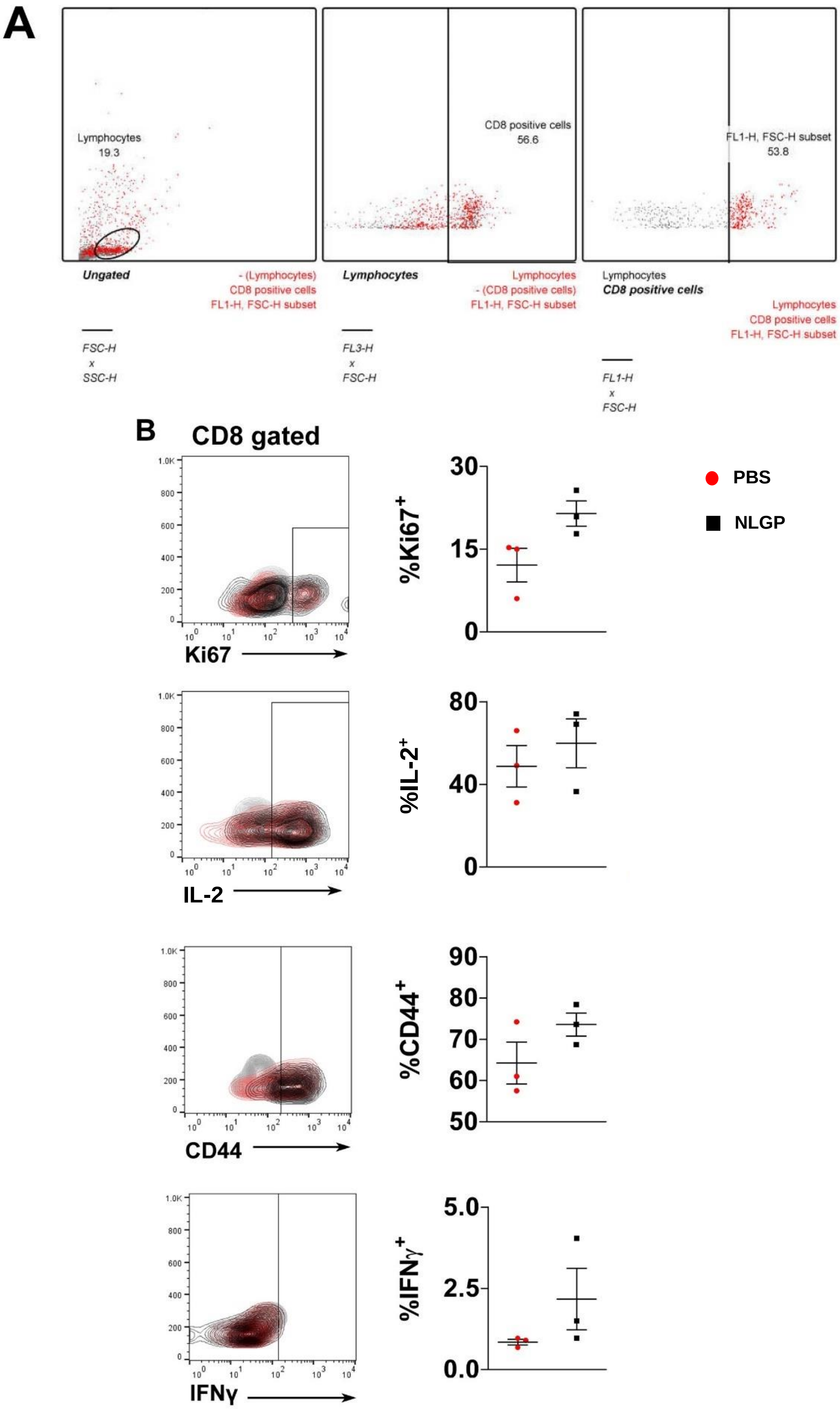

Figure S4B

Bhuniya et al

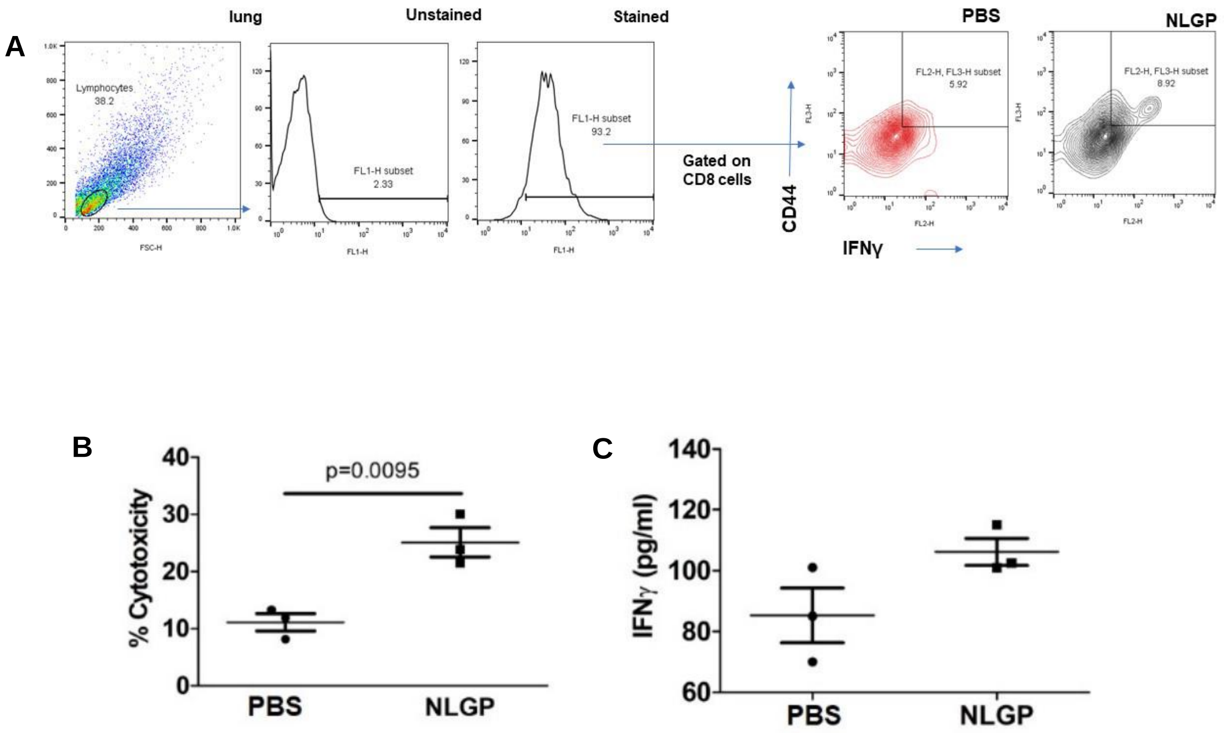

Figure S4C

Bhuniya et al

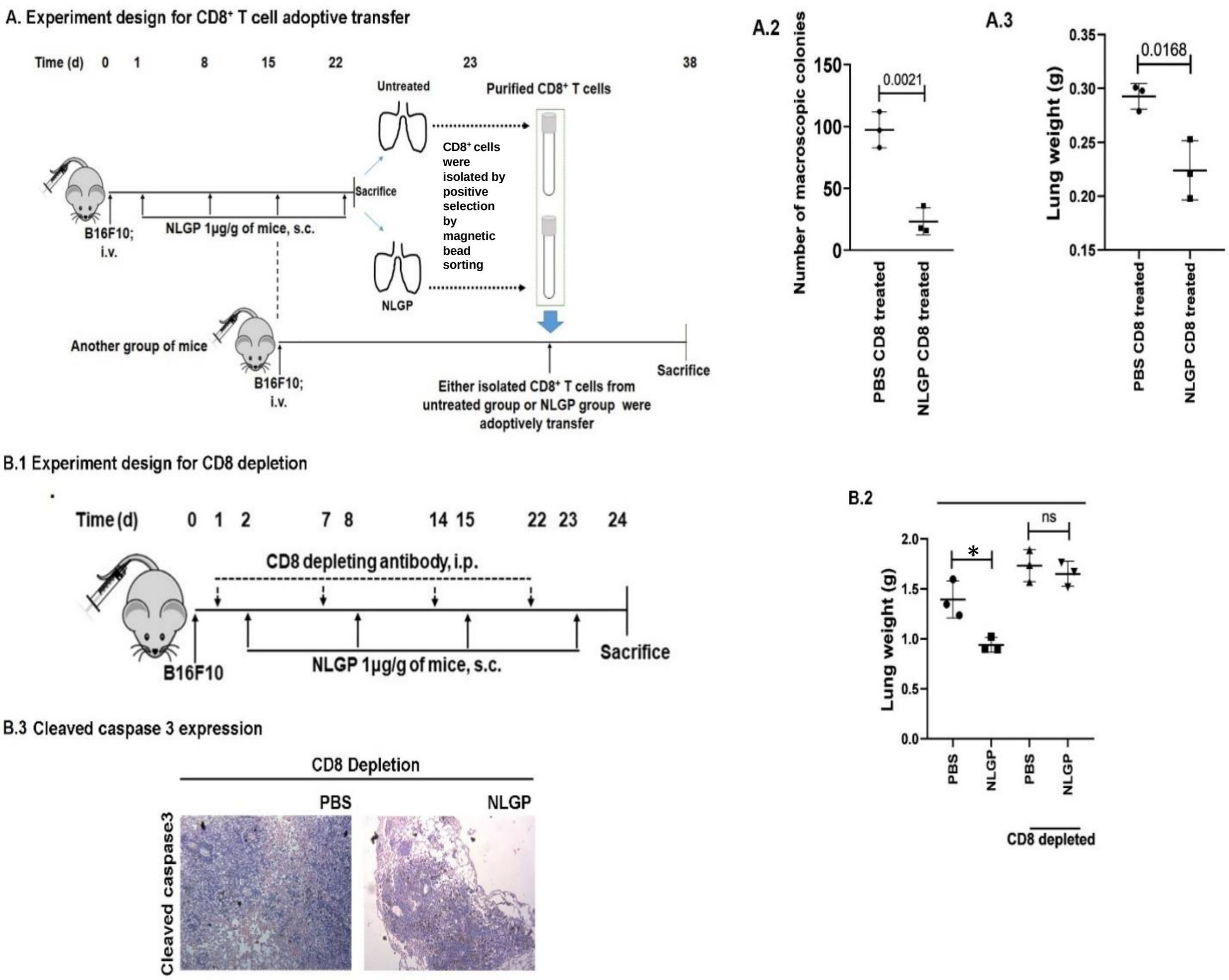

Figure S4D

Bhuniya et al

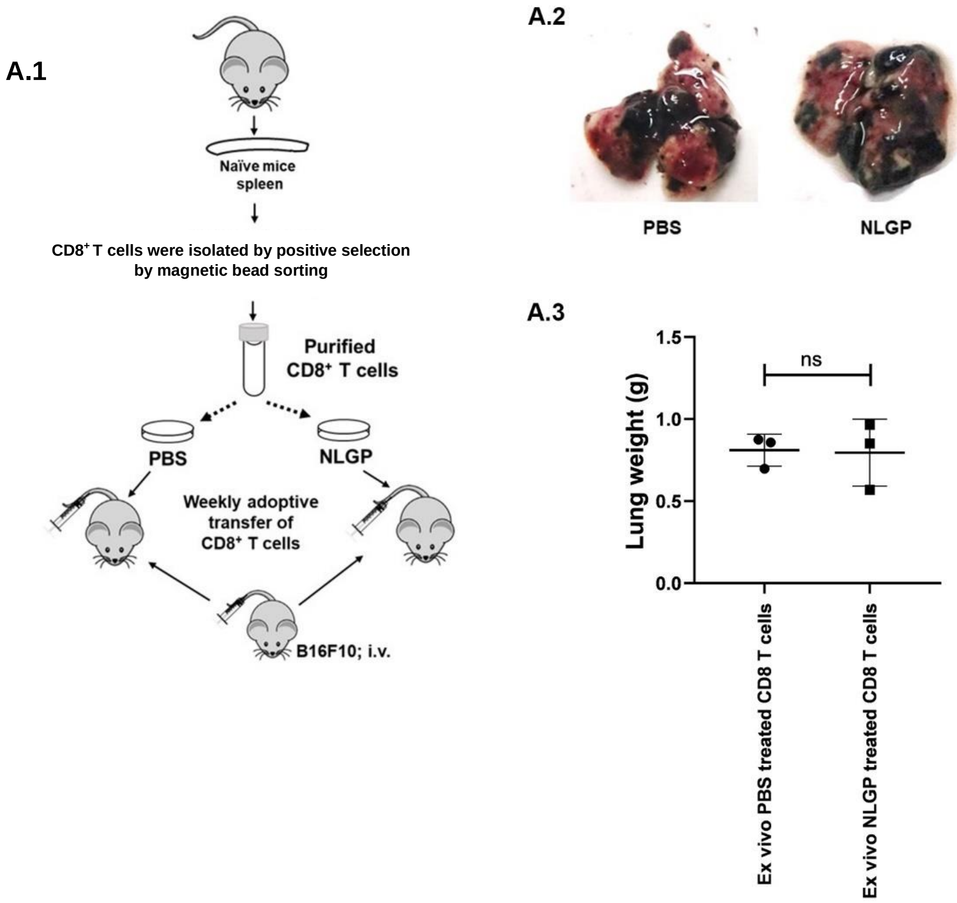

Figure S4E

Bhuniya et al

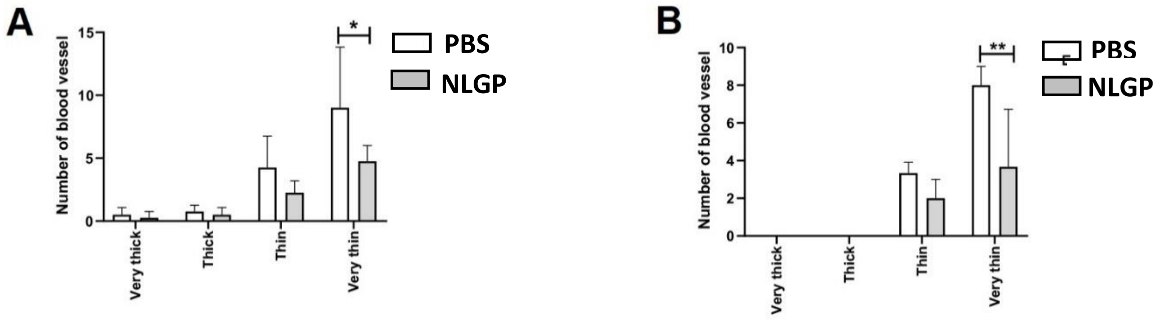

Figure S4F

Bhuniya et al

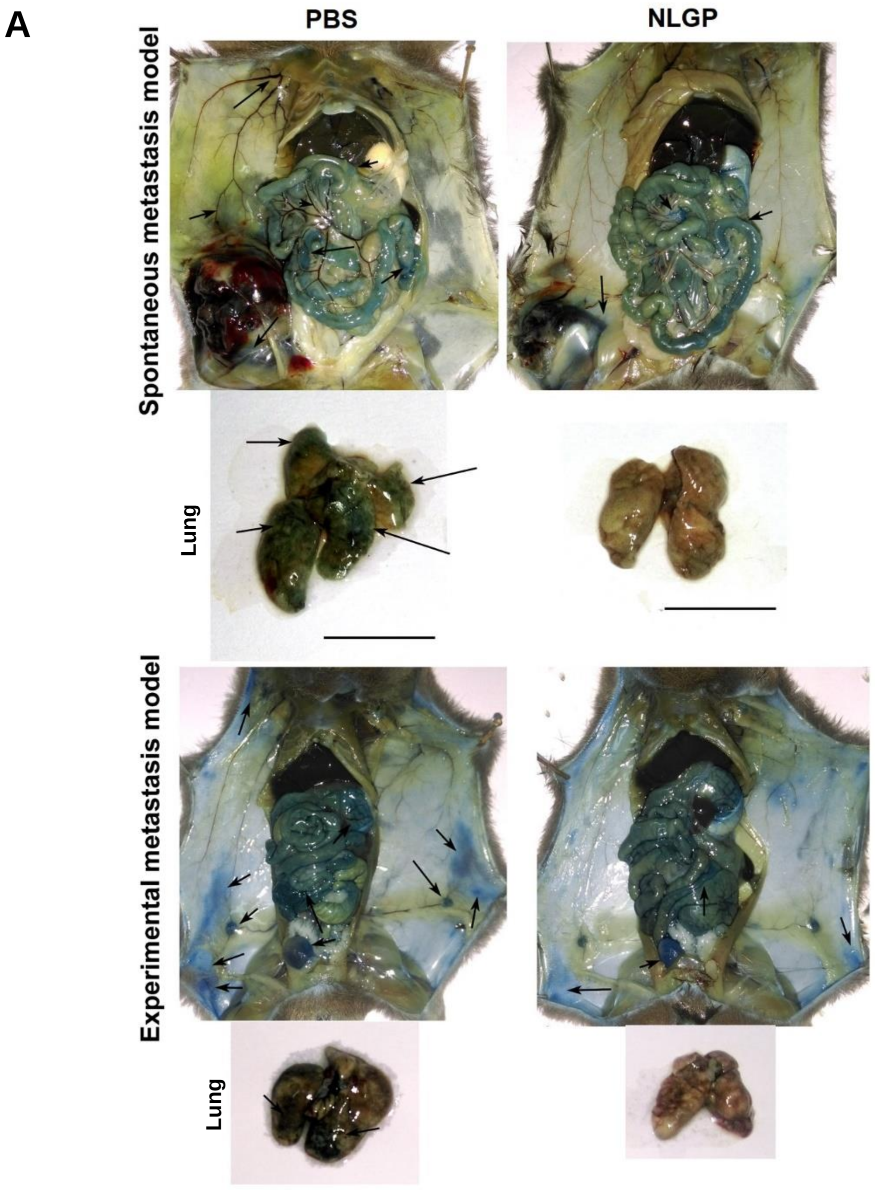

Figure S4G

Bhuniya et al

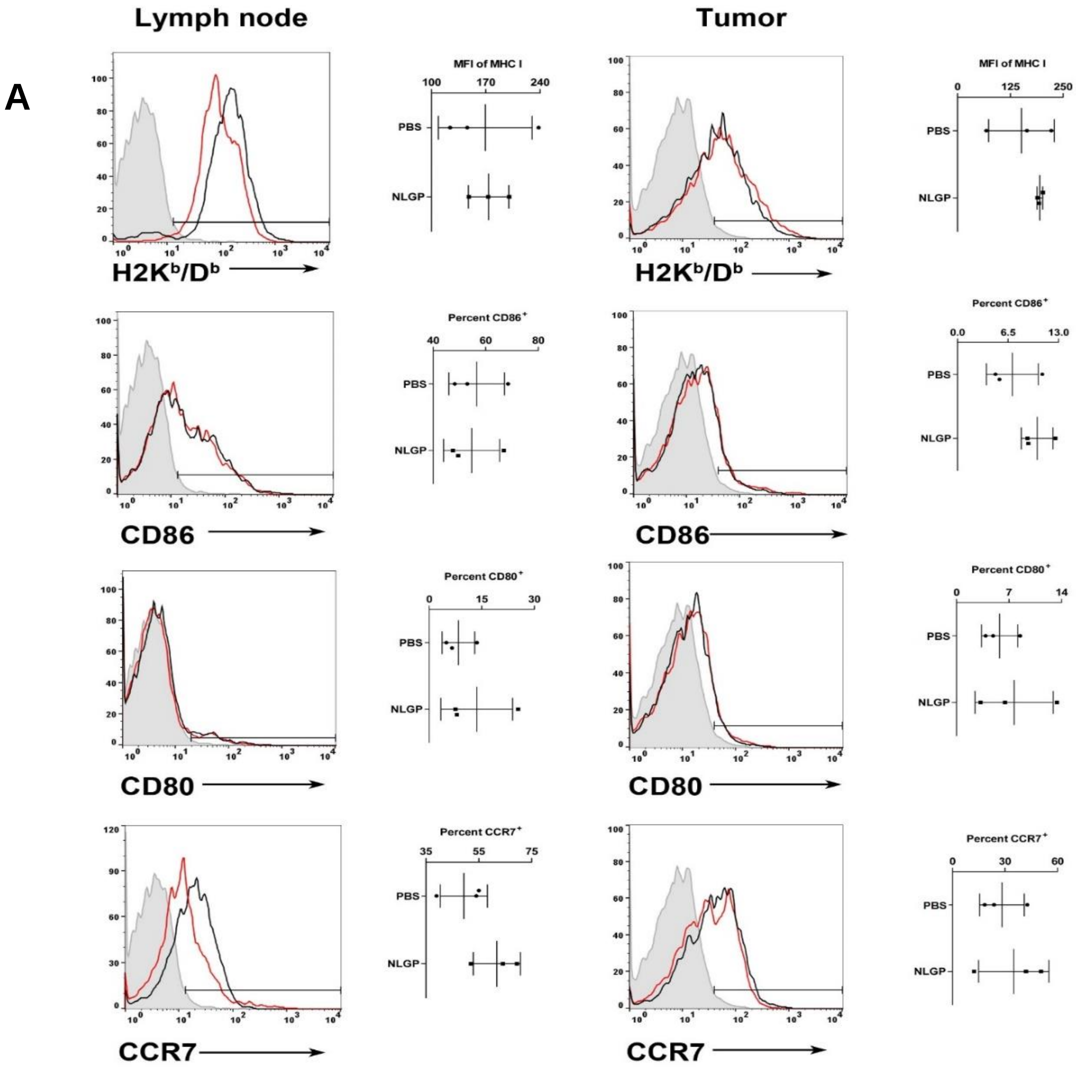

Figure S5A

Bhuniya et al

A

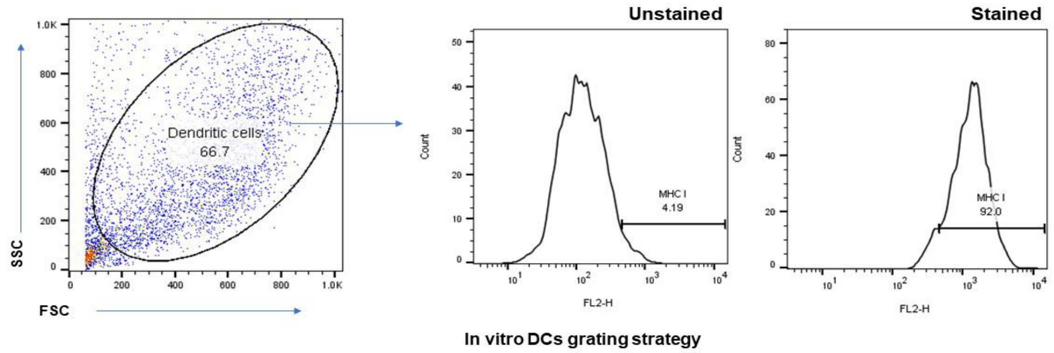

B

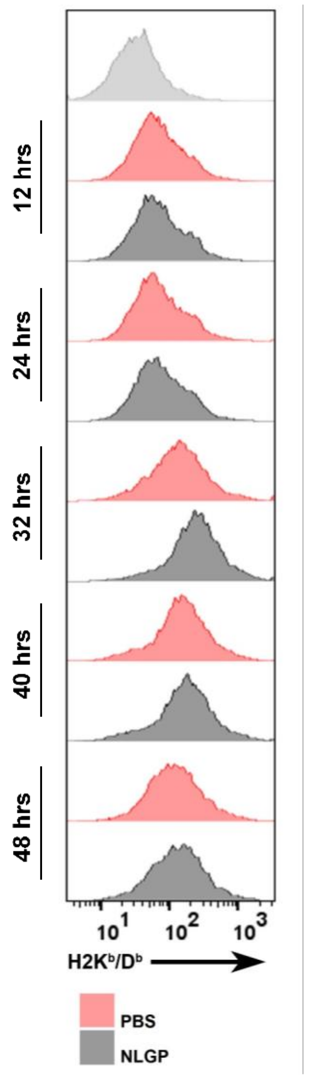

Figure S5B

Bhuniya et al

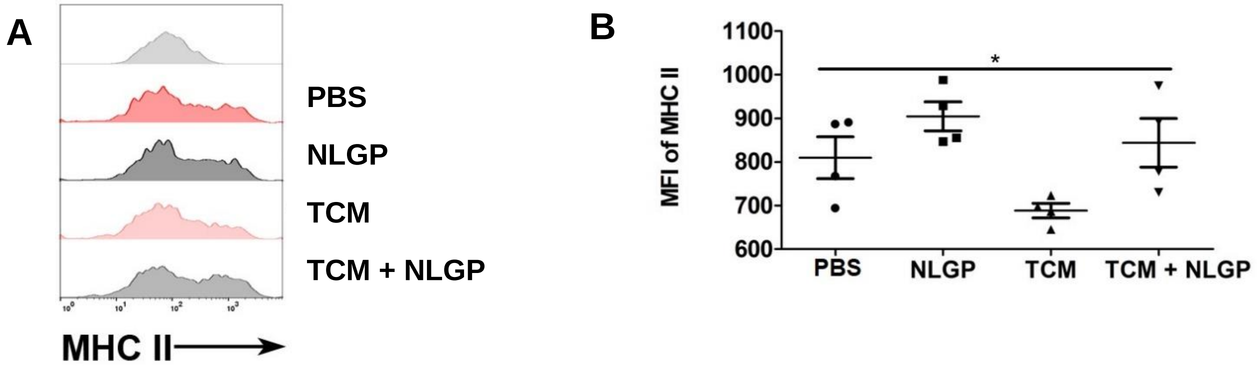

Figure S5C

Bhuniya et al

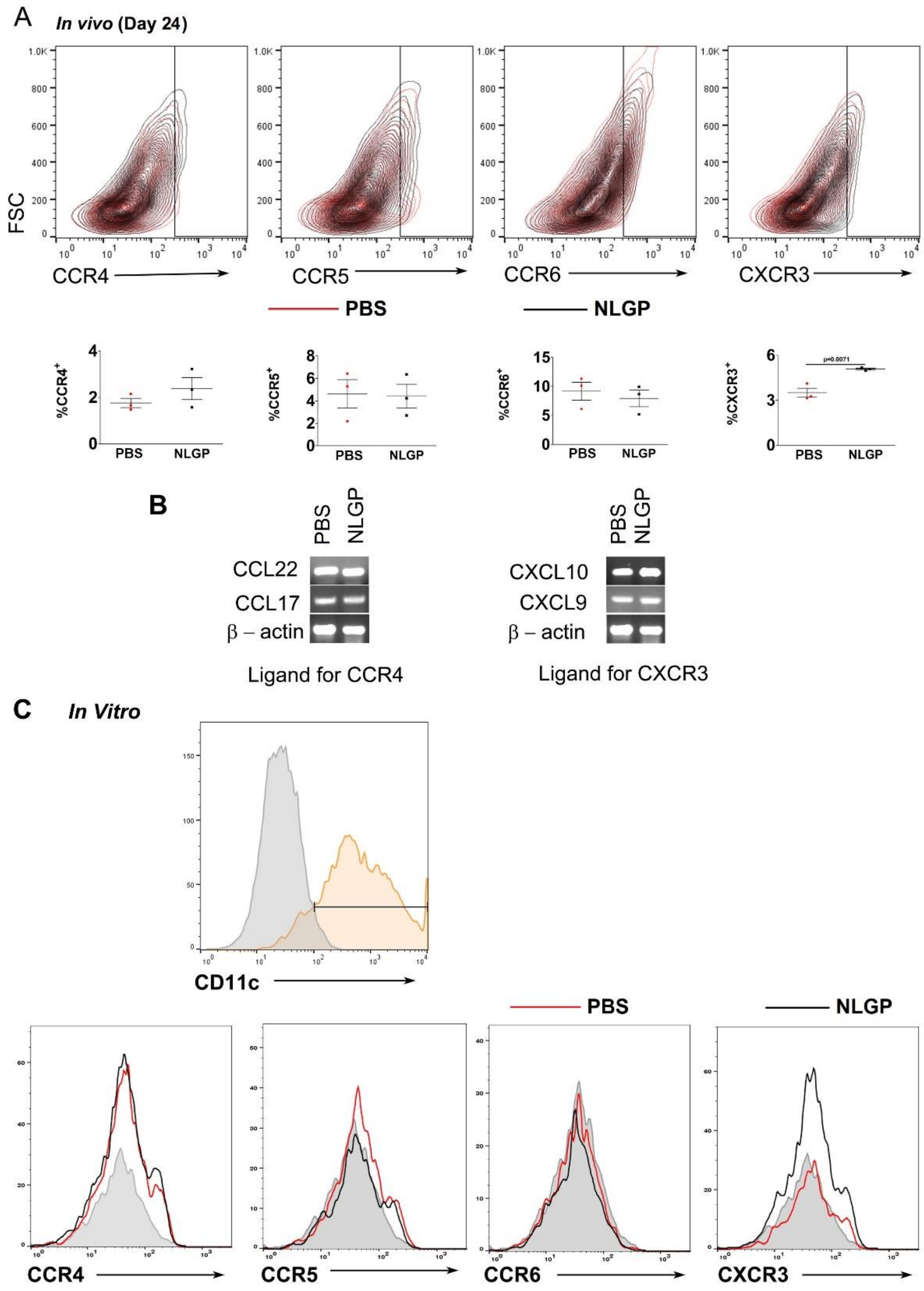

Figure S5D

Bhuniya et al

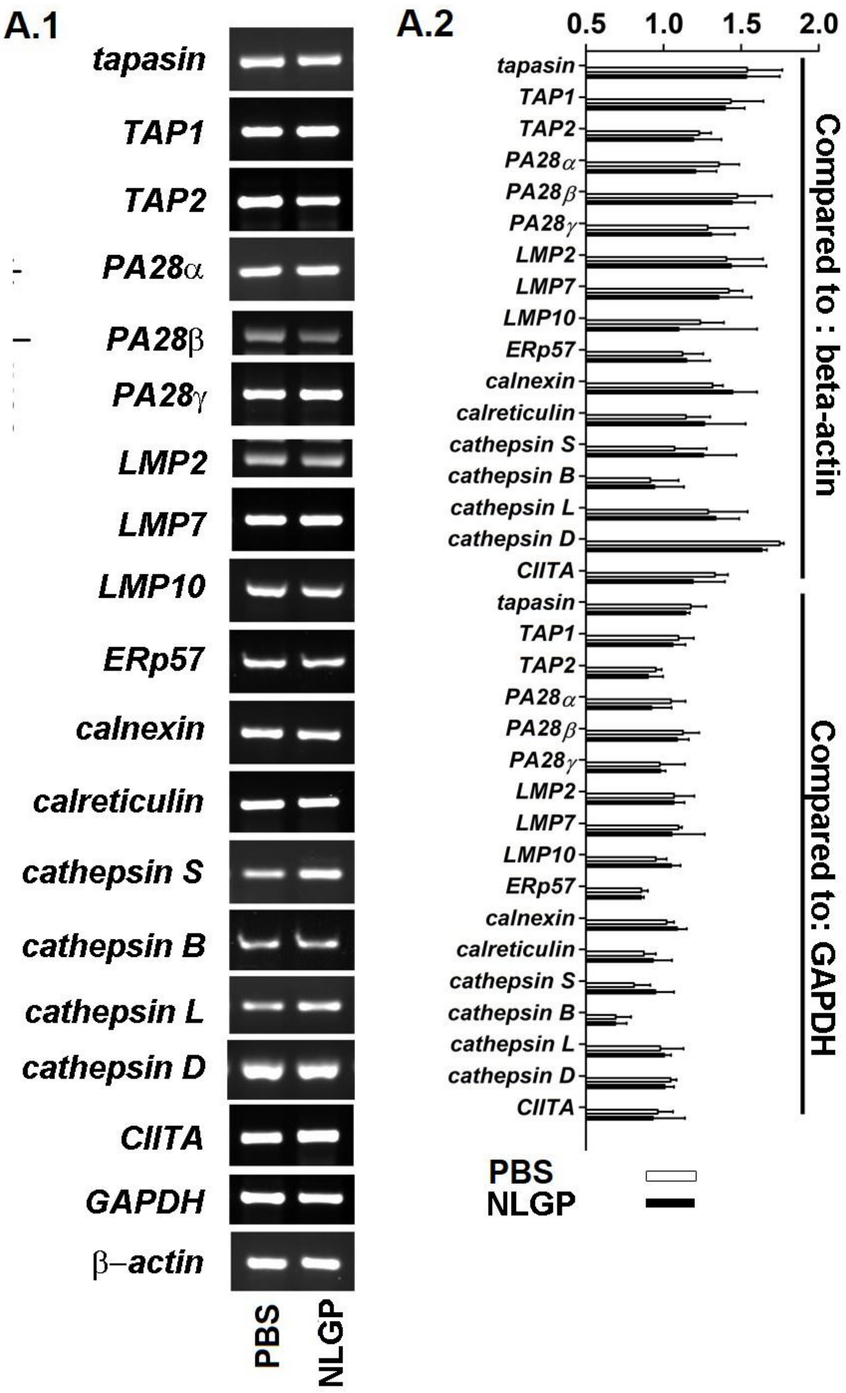

Figure S6A

Bhuniya et al

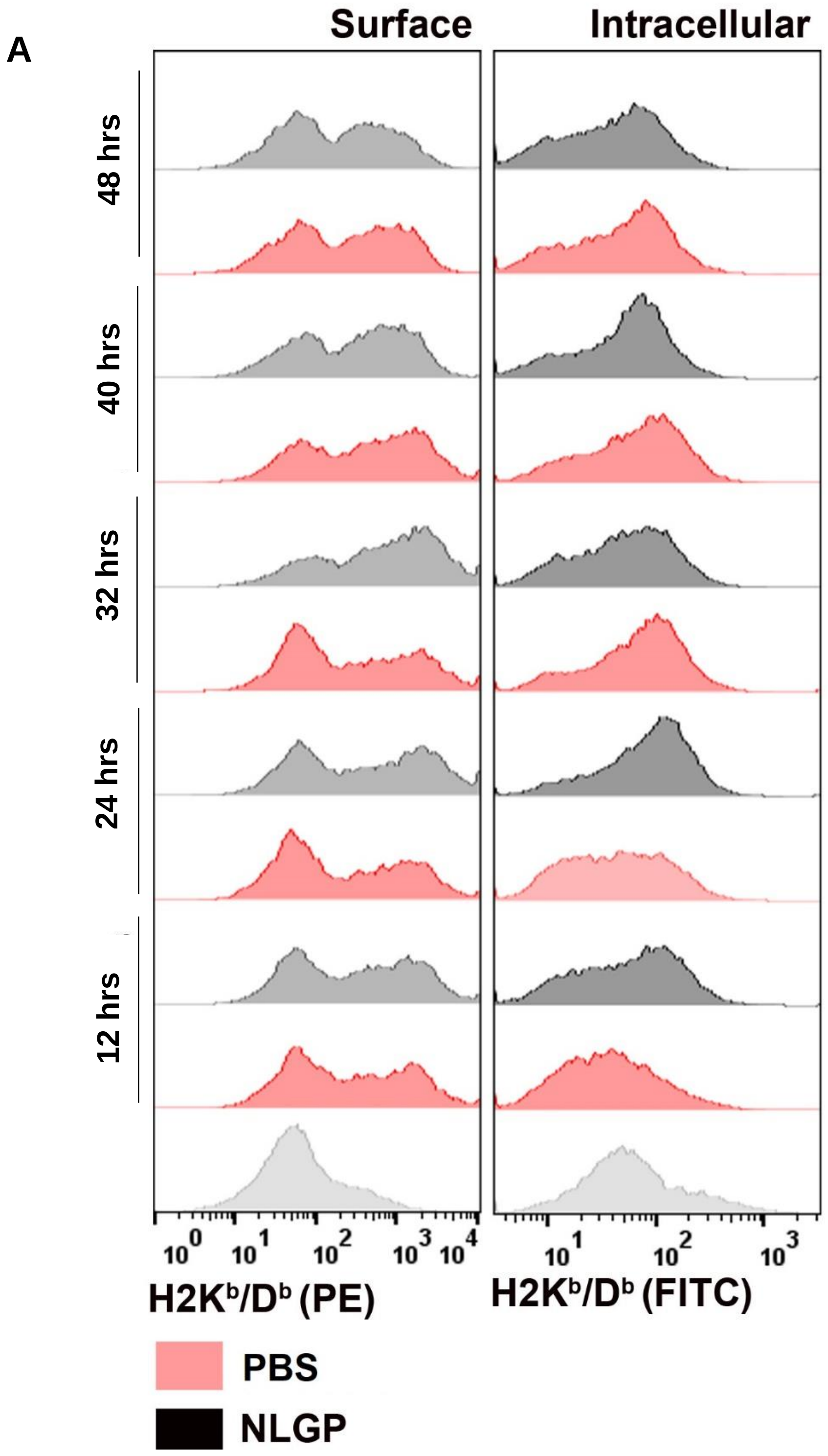

Figure S6B

Bhuniya et al

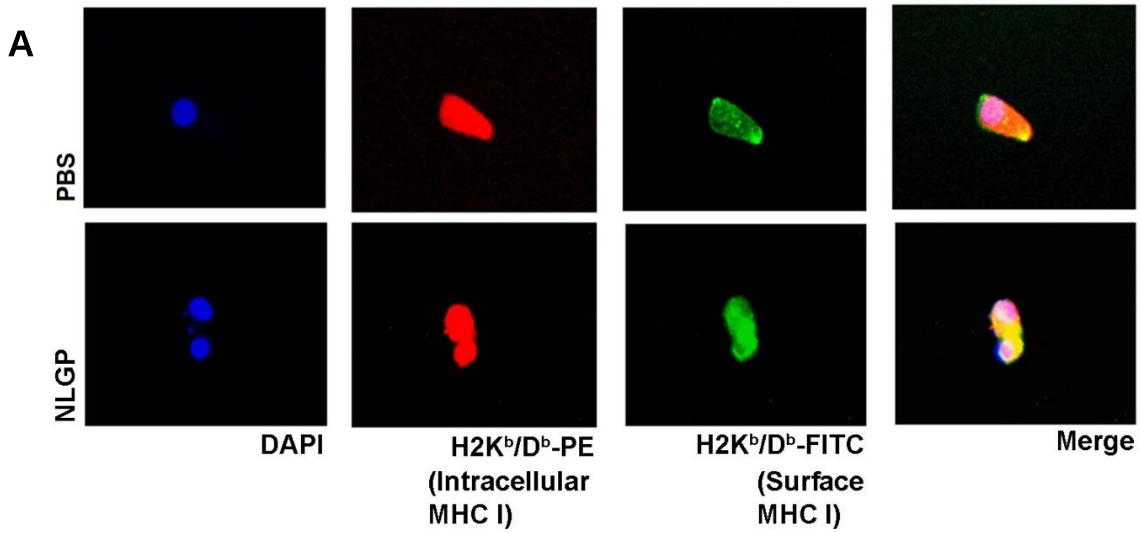

Figure S6C

Bhuniya et al

A

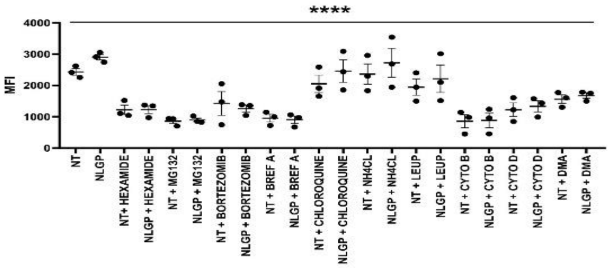

B

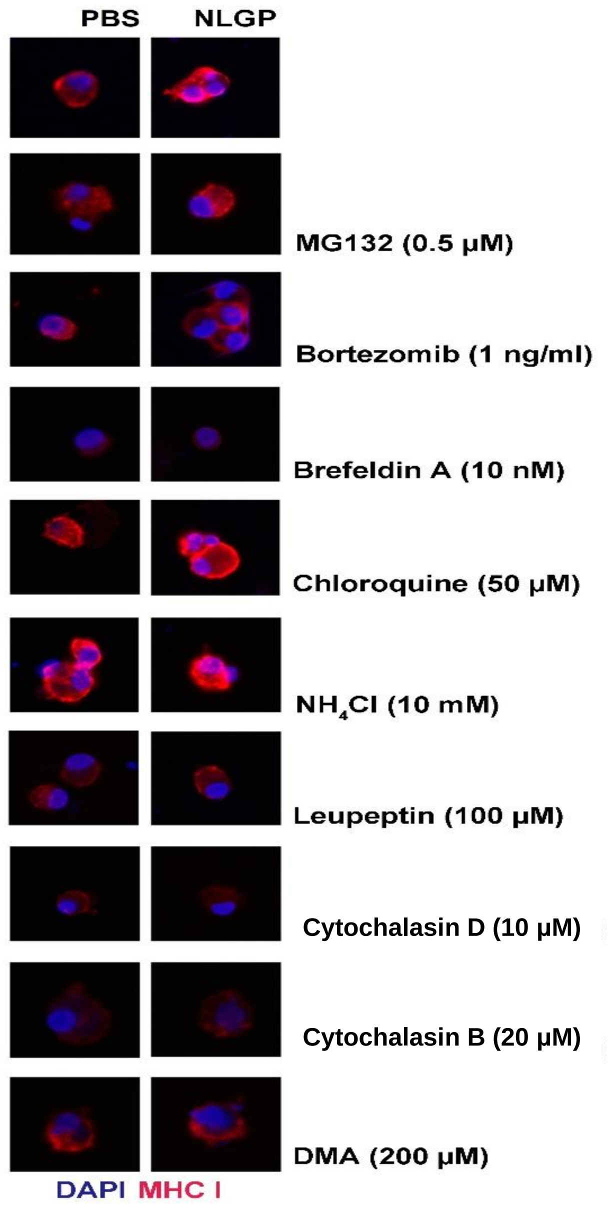

Figure S6D

Bhuniya et al

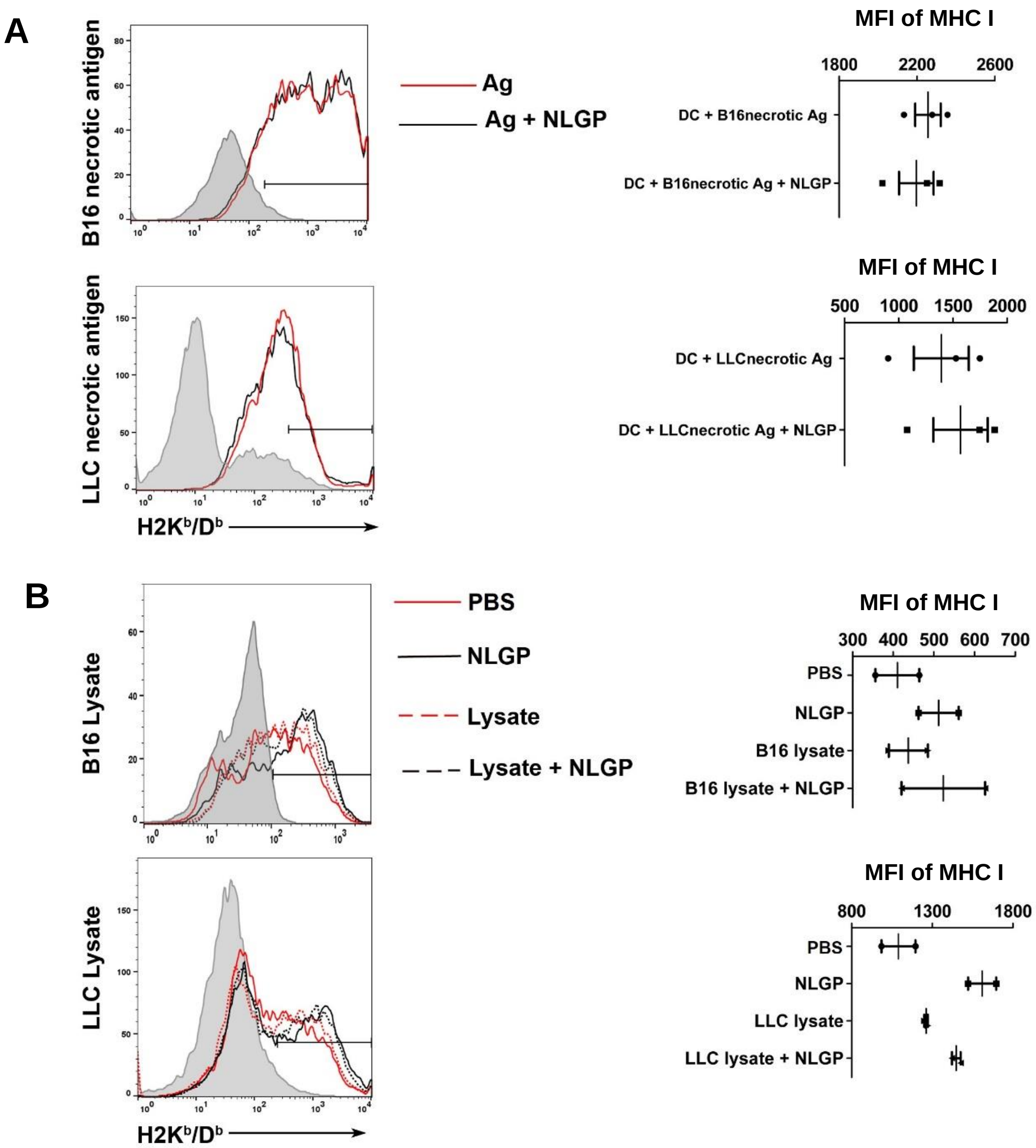

Figure S6E

Bhuniya et al

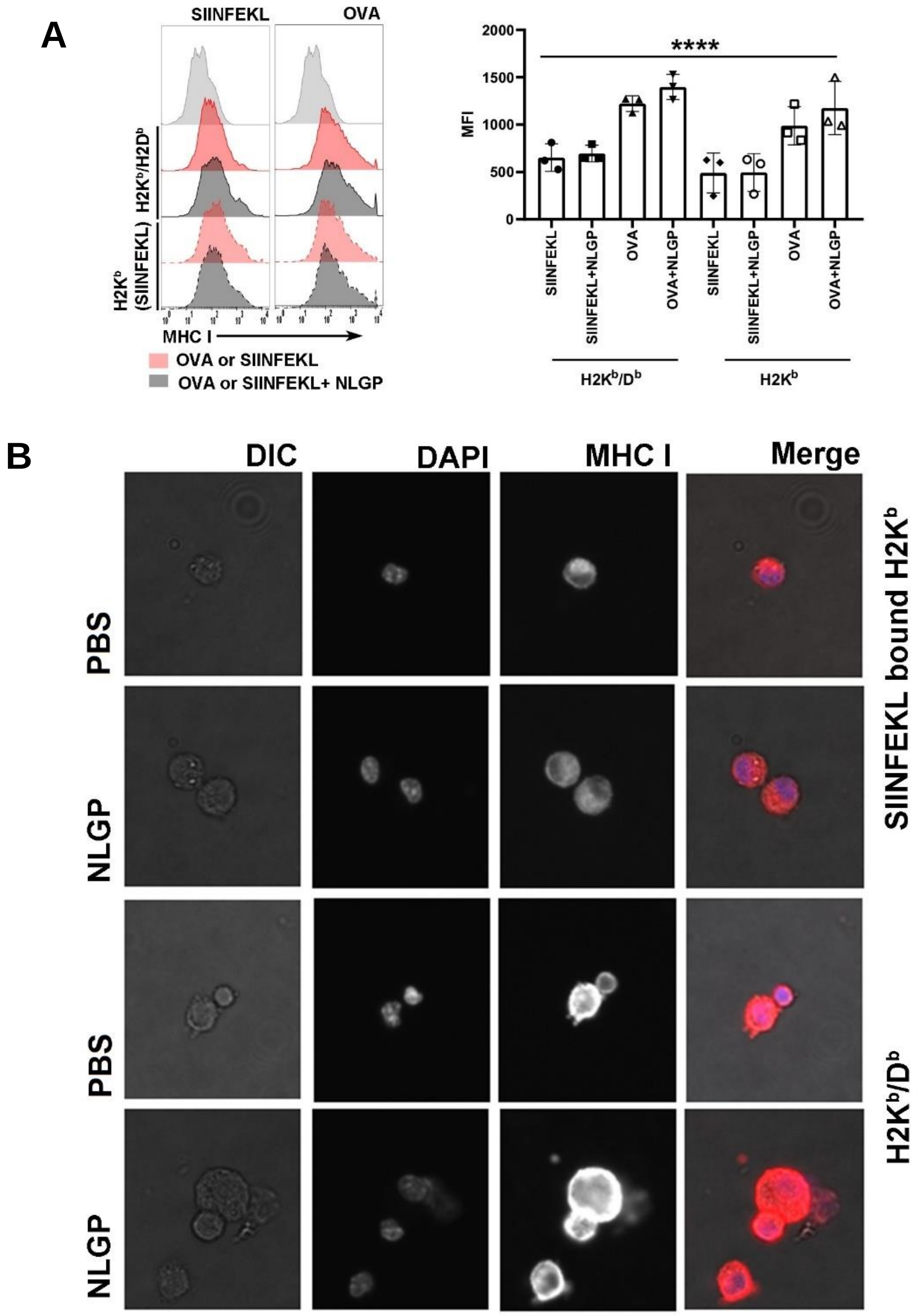

Figure S6F

Bhuniya et al

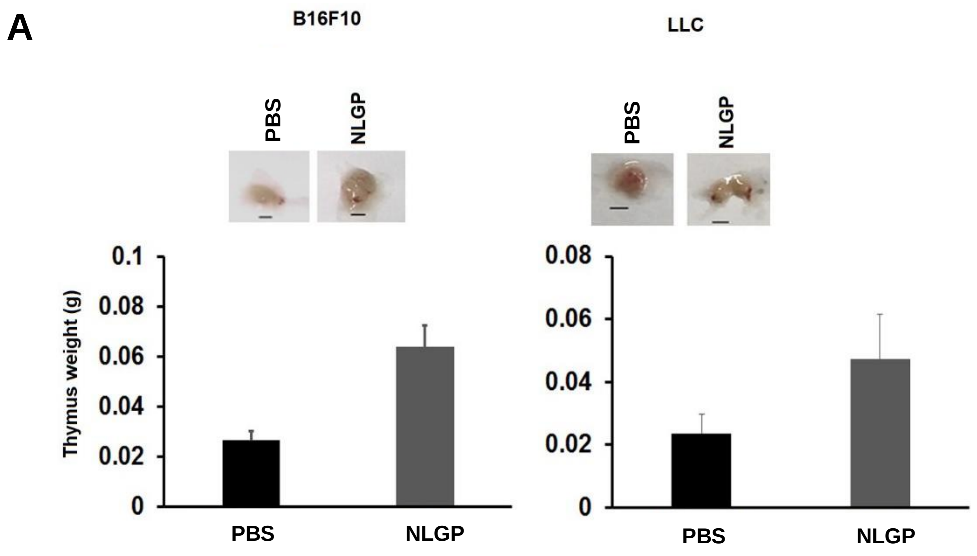

Figure S7

Bhuniya et al

Legends to Supplementary Figures

Figure S1. NLGP treatment reduces systemic metastasis. (A) Representative image of metastasis bearing organs from mice of PBS and NLGP treated groups. Black arrows indicate macroscopic metastasis. (B) Data showing number of macroscopic metastasis in mice from PBS and NLGP treated groups.

Figure S2. NLGP treatment reduces experimental metastasis. (A) Data showing number of macroscopic metastasis in mice from PBS and NLGP treated groups. (B.1, B.2) Kaplan-Meier survival analysis for B16F10 and LLC bearing mice respectively. Data were analyzed by Log-rank test. Mice number, median survival and statistical values are given in Tables in lower panel. (C.1) Representative diagram shows the gating strategy for cell cycle. (C.2) Representative diagram shows the gating strategy for Annexin V-PI. (C.3, C.4) Bar diagrams indicate mean percent positive cells ± SEM of cell death ($AnnV^{+}PI^{+}$), cell proliferation ($Ki67^{+}$) and cell cycle (PI^{+}) of mice from PBS and NLGP treated B16F10 and LLC cells respectively.

Figure S3. *In vitro* NLGP treatment reduces *in vivo* migration of tumor cells. (A) Bar diagrams indicate mean fluorescence intensity of $CFSE^{+}$ tumor cells ± SEM in lungs **(**B.1) Schematic representation of experimental design. B16F10 tumor cells were treated with NLGP (1.5 μg/ml) for 24 hrs followed by CFSE staining and inoculation into mice through t.v. Mice inoculated with B16F10 cells was treated with either PBS or NLGP (1 μg/g of body weight). After 6 hrs of NLGP treatment lungs were harvested. (B.2) Representative flow-cytometric dot plot shows $CFSE^{+}$tumor cells in *in vitro* NLGP and PBS treated groups. *In vitro* NLGP pre-treatment reduced the *in vivo* migration properties of tumor cells. (B.3) Bar diagrams indicate mean $CFSE^{+}$ cells ± SEM (B.4) Immunofluorescence analysis of $CFSE^{+}$ tumor cells in lung also show the same notion. (B.5) Bar diagrams indicate mean $CFSE^{+}$ cells (intensity ± SEM) in lung section (C)

Overnight serum starved B16F10 cells were seeded in the upper chamber of matrigel coated invasion chamber (BD-Corning) with or without NLGP against 10% serum gradient. After 24hrs of incubation, invaded cells were stained with 0.01% crystal violet and photographed under bright-field microscope. (C.2) Bar diagram shows mean invaded cells ± SEM. *In vitro* NLGP treatment prevents the invasion of tumor cells. (D) Tube-formation assay was performed on matrigel in presence or absence of NLGP.

Figure S4A. NLGP does not alter different cell populations in lung metastatic microenvironment of lungs. (A) Lungs were harvested from PBS and NLGP treated mice. Different cell populations were analyzed by flow-cytometrically on day dependent manner. Dot plots indicate mean percent positive cells ± SEM. One-way Anova was used for statistical analysis.

Figure S4B. NLGP treatment induces $CD8^+$ T cell activation of EMM mice. (A) Represents the gating strategies used for analysis. (B) $CD8^+Ki67^+$, $CD8^+CD44^+$, $CD8^+IL\text{-}2^+$, $CD8^+IFN\gamma^+$ cells were analyzed by flow-cytometry. Dot plots indicate mean positive percent cells ± SEM. Unpaired *t*-test was used for statistical analysis.

Figure S4C. NLGP treated $CD8^+$ T cells are more cytotoxic toward tumor cells. (A) Representative grating strategy for lung infiltrated $CD8^+$ T cells (B) $CD8^+$ T cells were isolated from PBS and NLGP treated mice lung by magnetic bead positive selection. Cytotoxic assay was performed against B16F10 cells according to manufacturer's protocol (see methods). Dot plot indicates mean cytotoxicity ± SEM (n=3). $CD8^+$ T cells from lungs of NLGP treated mice show greater cytotoxicity against B16F10 cells. (C) In other experiments, same $CD8^+$ T cells were co-cultured with 0.008% glutaraldehyde fixed B16F10 lysate pulsed BmDCs (BmDC:T=1:10). After 72hrs of co-culture IFNγ was measured by ELISA. Dot plot indicats mean IFNγ ± SEM (n=3).

Figure S4D. Schematic experimental design. (A) CD8$^+$ T cell adoptive transfer. Experimental design for the Figure 4D.1 (A.2) Dot plot indicates number of macroscopic colonies ± SEM on the lung surface after CD8$^+$ T cell adoptive transfer from PBS and NLGP treated mice lung. Data was analyzed by unpaired t-test. (A.3) Dot plot indicates lung weight ± SEM after CD8$^+$ T cell adoptive transfer from PBS and NLGP treated mice lung. Data was analyzed by unpaired t-test (B.1) Design for CD8$^+$ T cell depletion experiment; Figure 4D.2-D.3. (B.2) Representative immunohistochemistry was performed in paraffin embedded lung sections (5 μ) for cleaved caspase3 from CD8$^+$ T cells depleted PBS and NLGP treated mice. B.3 Dot plot indicates lung weight ± SEM after CD8$^+$ T cell depletion from PBS and NLGP treated mice lung. Data was analyzed by one-way Anova, followed by Turkey multiple comparison.

Figure S4E. *Ex vivo* NLGP treatment on CD8$^+$ T cells does not restrict metastasis. (A) Schematic diagram of *ex vivo* NLGP treatment. (B) Representative image of lung from CD8$^+$ T cells (*ex vivo* conditioned with PBS and NLGP) treated murine metastatic host. (C) Dot plot indicates mean lung weight ± SEM.

Figure S4F. NLGP treatment reduces neoangiogenesis. (A-B) Bar diagram indicates angiogenic vessels (number ± SEM) in spontaneous metastasis model from NLGP and PBS treated mice bearing B16F10 and LLC respectively. Vessels were counted according to the reference 13. Data was analyzed by two-way Anova followed by Turkey multiple comparison. *$p<0.05$; **$p<0.005$

Figure S4G. NLGP treatment reduces leakiness of blood vessels. (A) Evans-blue was inoculated into the PBS and NLGP treated mice of both SMM and EMM (see methods). After 30 minutes of evans-blue inoculation mice were sacrificed and macroscopic data was collected. Macroscopic observation exhibits greater blue coloration in PBS group, which indicates more

angiogenesis and simultaneous release of blue color in adjacent areas for leakiness (indicated by arrows). NLGP treated group shows less blue coloration in adjacent area indicating less leaky blood vessels.

Figure S5A. NLGP treatment alters DC properties. (A) Lymph nodes and tumors were harvested after single NLGP treatment and analyzed for MHC-I, CD80, CD86 and CCR7. Dot plot shows mean percent positive cells ± SEM (n=3). Data indicates slight improvement of all these parameters after NLGP treatment.

Figure S5B. NLGP optimizes MHC-I expression on 32hrs. (A) Representing gating strategy for BmDCs. (B) MHC-I expression was analyzed in PBS and NLGP treated BmDCs in time dependent manner. 32hrs was found to be optimum for MHC-I upregulation.

Figure S5C. NLGP treatment increases MHC-II under *in vitro* tumor condition. (A) BmDCs were cultured with and without TCM and NLGP. Histogram plot indicates the MHC-II expression. Dot plot indicates mean per cell expression ± SEM (n=3). NLGP treatment increases per cell expression of MHC-II in TCM condition. Data was analyzed by one-way Anova.

Figure S5D. NLGP induces CXCR3 and CCR4 homing receptor expression on T cells. (A) CCR4, CCR5, CCR6 and CXCR3 homing receptors were analyzed by flow-cytometry in T cells from PBS and NLGP treated mice. Dot plot indicates mean percent positive cells ± SEM (n=3). Data shows that NLGP induced expression of CXCR3 (more prominent) and CCR4 on lung T cells. (B) CCL22, CCL17 and CXCL10, CXCL9 expression levels were checked by RT-PCR in PBS and NLGP treated mice. No difference was detected in these chemokines. This observation also indirectly indicates that increased $CXCR3^{+}$and $CCR4^{+}$ T cells in NLGP treated lung is due to the systemic increase in $CXCR3^{+}$and $CCR4^{+}$ T cells in system. (C) 0.008% glutaladehyde fixed

B16F10 lysate pulsed BmDCs (with or without NLGP treated) were co-cultured with naïve splenic $CD8^+$ T cells (BmDC:T=1:10). Similar homing receptor was analyzed in these $CD8^+$ T cells and similar observation was obtained.

Figure S6A. NLGP does not alter expression of different genes in DCs. A.1 Representative agarose gel image of expression of different genes from PBS and NLGP treated BmDCs. A.2 Bar diagram indicates mean expression level ±SEM.

Figure S6B. NLGP induced increase in surface MHC-I expression does not proportionally reduces intracellular MHC-I expression. (A) Surface and intracellular MHC-I expression was measured in PBS and NLGP treated BmDCs in time dependent manner. As data shows there is no proportional decrease in intracellular MHC-I with increase in surface MHC-I, NLGP may induces MHC-I translation.

Figure S6C. NLGP treatment increases MHC-I expression on DCs. (A) Surface and intracellular MHC-I expressions were studied by immunofluorescence. Surface expression was measured by PE-tagged antibody, whereas intracellular expression was measured by FITC tagged antibody.

Figure S6D. Chloroquine and NH_4Cl augment NLGP induced MHC-I expression. (A) Dot plot indicates mean MFI ± SEM of MHC I molecules in presence of inhibitors (n=3). Data was analyzed by one-way Anova. (B) Representative image shows the surface MHC-I expression as detected by immunofluorescence microscopy in presence of different inhibitors of antigen processing and presentation pathway.

Figure S6E. NLGP does not augment MHC-I expression in presence of antigen. BMDCs were treated with different types of antigens in presence or absence of NLGP. (A) Histogram

shows representative data of necrotic antigen. Dot plot indicates mean per cell expression of MHC I ± SEM (n=3). (B) Histogram shows representative data of soluble antigen. Dot plot indicates mean per cell expression of MHC I ± SEM (n=3).

Figure S5I. Surface expression of MHC-I in presence of OVA, SIINFEKL and NLGP. (A) BmDCs were treated with either OVA or SIINFEKL in presence or absence of NLGP and expression of surface MHC-I was measured by $H2K^b/D^b$ and SIINFEKL bound MHC-I specifie H2Kb antibody. Dot plot indicates mean MFI ± SEM of surface MHC I expression after treatment with OVA or SIINFEKL in presence or absence of NLGP. MHC I expression was detected by two different antibodies of MHC-I clone. (B) Surface MHC-I expression was analyzed by immunofluorescence microscope after treatment with OVA or SIINFEKL. Two separate antibodies (Clone -25-D1.16, specifically detect SIINFEKL bound $H2K^b$ and clone – 28-8-6, detect $H2K^b/D^b$) were used.

Figure S7. NLGP prevents thymus atrophy. Representative figure shows the image of thymus in PBS and NLGP treated mice. Bar diagram indicates mean thymus weight ± SEM (n=3).

Table S1: Primer list

Sr. No.	gene	Chromosome	Primer sequence (5'-3')	Primer length	Product size	Tm	No. of cycle
1	Beta actin F	Choromosome 1 NC_000068.7	5'-CAACCGTGAAAAGATGACCC-3'	20	228bp	54.0	28
	Beta actin R		5'-ATGAGGTAGTCTGTCAGGTC-3'	20		53.1	
2	Beta actin F	Choromosome 1 NC_000068.7	5'-GGCTGTATTCCCCTCCATCG-3'	20	154bp	57.6	28
	Beta actin R		5'-CCAGTTGGTAACAATGCCATG-3'	21		55.9	
3	Beta actin F	Choromosome 1 NC_000068.7	5'-CCTCTATGCCAACACAGTGC-3'	20	206bp	62.5	28
	Beta actin R		5'-CCTGCTTGCTGATCCACATC-3'	20		62.5	
4	GAPDH F	Chromosome 6 NC_000072.6	5'-GTTGTCTCCTGCGACTTCA-3'	19	184bp	54.8	28
	GAPDH R		5'-GGTGTTCCAGGGTTTCTTA-3'	19		54.1	
5	VEGF F	Chromosome 17, NC_000083.6	5'-GGACCCTGGCTTTACTGCTG-3'	20	201bp	63.7	28
	VEGF R		5'CACAGGACGGCTTGAAGATG-3'	20		63.7	
6	TGFβ F	Chromosome 7, NC_000073.6	5'-TGCGCTTGCAGAGATTAAAA-3'	20	197bp	53.2	28
	TGFβ R		5'-GCTGAATCGAAAGCCCTGTA-3'	20		57.3	
7	Granzyme B F	Chromosome 14, NC_000080.6	5'-TCGACCCTACATGGCCTTAC-3'	20	198bp	59.4	28
	Granzyme B R		5'- ATGGTAAAATGCATTCCCCA -3'	20		53.2	
8	IFNγ F	Chromosome 10, NC_000076.6	5'-ACTGGCAAAAGGATGGTGAC-3'	20	227bp	57.3	28
	IFNγ R		5'-TGAGCTCATTGAATGCTTGG-3'	20		57.3	
9	HIF1a F	Chromosome 12, NC_000078.6	5'-TCAAGTCAGCAACGTGGAAG-3'	20	198bp	57.3	28
	HIF1a R		5'-TATCGAGGCTGTGTCGACTG-3'	20		59.4	
10	CD31 F	Chromosome 11, NC_000077.6	5'-AGCCCACCAGAGACATGGAA-3'	20	337bp	59.4	28
	CD31 R		5'-CTGGCTCTGTTGGAGGCTGT-3'	20		61.4	
11	VEGFR2 F	Chromosome 5, NC_000071.6	5'-ACAGACAGTGGGATGGTCC-3'	19	271bp	58.8	28
	VEGFR2 R		5'-AAACAGGAGGTGAGCGCAG-3'	19		58.8	
12	H2Kb F	Chromosome 17, NC_000083.6	5'- AGTGGTGCTGCAGAGCATTACAA -3'	23	153bp	64.2	28
	H2Kb R		5'- CCCAGATCTAAAGGTGAAGTCACC -3'	24		63.6	
13	H2Db F	Chromosome 17, NC_000083.6	5'- GCTGGTGAAGCAGAGAGACTCA -3'	22	153bp	63	28
	H2Db R		5'- GGTGACTTTATCTTCAGGTCTGCT -3'	24		65.2	
14	B2m F	Chromosome 2, NC_000068.7	5'- ATGGGAAGCCGAACATACTG -3'	20	177bp	58.4	28
	B2m R		5'- CAGTCTCAGTGGGGGTGAAT -3'	20		60.5	
15	LMP2 F	Chromosome 17, NC_000083.6	5'- CACCACAGATGCCATCACTC -3'	20	156bp	59.4	28
	LMP2 R		5'- GGACTTCTGGGGATCAGTCA -3'	20		59.4	
16	LMP 7 F	Chromosome 17, NC_000083.6	5'- CGGGACAGATGTTTTCCACT -3'	20	197bp	57.3	28
	LMP7 R		5'- CACTTTCACCCAACCGTCTT -3'	20		57.3	
17	LMP10 F	Chromosome 8, NC_000074.6	5'- CAGAGCCAAGGGCAGTAAAG -3'	20	168bp	59.4	28
	LMP10 R		5'- GTGATCACACAGGCATCCAC -3'	20		59.4	
18	PA28a F	Chromosome 14, NC_000080.6	5'- AAGCCAAGGTGGATGTGTTC -3'	20	167bp	57.4	28
	PA28a R		5'- GGGTACTGGGATGTCCAATG -3'	20		59.4	
19	PA28b F	Chromosome 14, NC_000080.6	5'- GGGGTACTACTCACGGTGGA -3'	20	176bp	61.4	28
	PA28b R		5'- CTGGGATAGGGATGTCCAGA -3'	20		59.4	
20	PA28g F	Chromosome 11, NC_000077.6	5'- CCAGCAGCTTGTGGACATTA -3'	20	191bp	57.3	28
	PA28g R		5'- GGTAAGATGCGGCTTCACTC -3'	20		59.4	
21	TAP1 F	Chromosome 17, NC_000083.6	5'- AGACTGACAAGGCCTCTGGA -3'	20	197bp	59.4	28
	TAP1 R		5'- AGGTACCTGAAACCCCGTCT -3'	20		59.4	
22	TAP2 F	Chromosome 17, NC_000083.6	5'- AAGGTGGTGGGGCTCTACTT -3'	20	245bp	59.4	28
	TAP2 R		5'- GCCTCCTTGTAGTGGCTGAC -3'	20		61.4	
23	ERp57 F	Chromosome 2, NC_000068.7	5'-ATTCCGTCCATTACATCTTG-3'	20	170bp	53.2	28
	ERp57 R		5'-TGAGTAAGTCCTTGCCTTGT-3'	20		55.3	
24	Tapasin F		5'- TACGGTACACCTGCCCTACC -3'	20	188bp	61.4	28

	Tapasin R	Chromosome 17, NC_000083.6	5'- CTCTGAGCTCCCACTTGACC -3'	20		61.4	
25	Calnexin F	Chromosome 11, NC_000077.6	5'- GGCTAGACGACGAACCTGAG -3'	20	187bp	61.4	28
	Calnexin R		5'- AGGCTTCCATTTGCCCTTAT -3'	20		55.3	
26	Calreticulin F	Chromosome 8, NC_000074.6	5'- AGACGTGGGGTGTTACCAAG-3'	20	224bp	59.4	28
	Calreticulin R		5'-TGGCCTCTACAGCTCATCCT-3'	20		59.4	
27	Cathepsin S F	Chromosome 3, NC_000069.6	5'-ATGATTCACAATCTCGAATA-3'	20	152bp	49.1	28
	Cathepsin S R		5'-TATGACCTGAAAGTGACAGT-3'	20		53.2	
28	Cathepsin D F	Chromosome 7, NC_000073.6	5'-TTACTCAAAAACTACCTGGA- 3'	20	168bp	51.2	28
	Cathepsin D R		5'-ACTGTTGTACTTATGGTGGA-3'	20		53.2	
29	Cathepsin L F	Chromosome 13, NC_000079.6	5'-TGACACAGGGTTCGTGGATA-3'	20	162bp	57.3	28
	Cathepsin L R		5'-TCGAGGTTCTTGCTGCTACA-3'	20		57.3	
30	Cathepsin B F	Chromosome 14, NC_000080.6	5'-TCTACAATTCTCATGTAGGC-3'	20	176bp	53.2	28
	Cathepsin B R		5'-GTAGGAAGTGTACCCAAAGT-3'	20		55.3	
31	CD86 F	Chromosome 16, NC_000082.6	5'-CAAGAGTTTCCATCTCCTCA-3'	20	197bp	58.4	25
	CD86 R		5'-GGTTCAAGTTCCTTAAGGTT-3'	20		56.3	
32	CD80 F	Chromosome 16, NC_000082.6	5'-GGCTCATTCTTCTCTTTGTG-3'	20	204bp	58.4	28
	CD80 R		5'-ACACTTTTAGTTTCCCAGCA-3'	20		58.4	
33	CCR7 F	Chromosome 11, NC_000077.6	5'-GTGTGCTTCAAGAAGGATGT-3'	20	201bp	55.3	28
	CCR7 R		5'-GAAGGGAAGAATTAGGAGGA-3'	20		55.3	
34	CIITA F	Chromosome 16, NC_000082.6	5'-ACACCTGGACCTGGACTCAC-3'	20	229bp	61.4	28
	CIITA R		5'-GCTCTTGGCTCCTTTGTCAC-3'	20		59.4	
35	CCL 17 F	Chromosome 8, NC_000074.6	5'-ATGAGGTCACTTCAGATGCT-3'	20	148bp	55.3	28
	CCL 17 R		5'-AGCTCACCAACTTCCTGATA-3'	20		55.3	
36	CCL 22 F	Chromosome 8, NC_000074.6	5'-CTGATGCAGGTCCCTATGGT-3'	20	237bp	60.5	28
	CCL 22 R		5'-CATGGTCATCAGGTCCTCCT-3'	20		60.5	
37	CXCL 9 F	Chromosome 5, NC_000071.6	5'-TGGGCATCATCTTCCTGGAG-3'	20	204bp	60.5	28
	CXCL 9 R		5'-CCGGATCTAGGCAGGTTTGA-3'	20		60.5	
38	CXCL 10 F	Chromosome 5, NC_000071.6	5'-CCAAGTGCTGCCGTCATTTT-3'	20	177bp	58.4	28
	CXCL 10 R		5'-CTCAACACGTGGGCAGGATA-3'	20		60.5	
